# Complete genome sequence, metabolic profiling and functional studies reveal *Ligilactobacillus salivarius LS-ARS2* is a promising biofilm-forming probiotic with significant antioxidant, antibacterial, and antibiofilm potential

**DOI:** 10.3389/fmicb.2025.1535388

**Published:** 2025-03-20

**Authors:** Sinjini Patra, Biswaranjan Pradhan, Anasuya Roychowdhury

**Affiliations:** ^1^Biochemistry and Cell Biology Laboratory, School of Basic Sciences, Indian Institute of Technology Bhubaneswar, Odisha, India; ^2^S. K. Dash Center of Excellence of Biosciences and Engineering & Technology (SKBET), Indian Institute of Technology Bhubaneswar, Odisha, India

**Keywords:** probiotics, microbial dysbiosis, oxidative stress, whole genome sequencing analysis, biofilm formation and anti-biofilm potential, metabolic profile analysis, food supplement, biotherapeutic

## Abstract

**Background:**

Probiotics restore microbial balance and prevent gut-inflammation. Therefore, finding out novel probiotic strains is a demand. As gut-microbe, benefits of *Ligilactobacillus salivarius (LS)* are established. However, strain-specific detailed studies are limited. Here, we illustrate probiotic attributes of novel *LS-ARS2* for its potential application as food-supplement and/or therapeutic to improve gut-health.

**Methods:**

Whole genome sequencing (WGS) and phylogenetic analysis confirm the strain as *LS*. To establish probiotic properties, acid-bile tolerance, auto-aggregation, cell-surface-hydrophobicity, biofilm-formation, and adhesion-assays are performed. To ensure safety attributes, antibiotic-susceptibility, hemolytic, DNase, trypan-blue, and MTT assays are done. ABTS, DPPH, superoxide, hydroxyl free radical scavenging assays are used to determine anti-oxidant potential. Antibacterial assays, including co-culture assay with pathogen and pathogenic biofilm-inhibition assays, are performed to explore antibacterial efficacy. To characterize metabolic-profile of *LS-ARS2*-derived cell-free-supernatant (CFS), HRMS analysis are carried out. Consequently, WGS-analyses predict potential molecular associations related to functional outcomes.

**Results:**

We find *LS-ARS2* a remarkable fast-growing strain that shows acid and bile tolerance (>60% survival rate), indicating promising gut-sustainability. High auto-aggregation capacity (>80%), robust cell-surface hydrophobicity (>85%), and adhesion efficacy to Caco-2 cells illustrate significant potential of *LS-ARS2* for gut colonization. Fascinatingly, *LS-ARS2* is able to form biofilm within 24 h (*p* < 0.0001), rare among *LS* strains, indicating the potential of the strain for efficient stay in the gut. The strain ensures safety attributes. *LS-ARS2*-WGS analysis recognizes probiotic-specific determinants, predicts genomic stability, identifies orthologous-clusters for diverse functions, and predicts metabolites and bacteriocins. HRMS-studies with *LS-ARS2-*CFS further validate the presence of diverse beneficial metabolites with antimicrobial and immunomodulatory potential. *LS-ARS2* shows significant antioxidant properties in ABTS (>60%), DPPH (>10 U/mL), superoxide (>70%), and hydroxyl free radical scavenging assays (>70%). Further, *LS-ARS2* shows antimicrobial activities against Gram-positive Methicillin-resistant *Staphylococcus aureus (MRSA)* and Gram-negative multidrug-resistant clinical strains enterotoxigenic *Escherichia coli, Vibrio cholerae,* and *Shigella flexneri*. Anti-*Salmonella* effect of *LS-ARS2* is prominent (*p* < 0.0001). Most interestingly, *LS-ARS2*-CFS inhibits *MRSA*-biofilm (*p* < 0.0001), again rare among *LS* strains.

**Conclusion:**

*LS-ARS2* is a novel, fast-growing, biofilm-forming probiotic with significant antioxidant, antibacterial, and anti-biofilm potentials, suggesting the promising applications of *LS-ARS2* for combating pathogenic biofilms and improving gut-health. However, further *in vivo* studies would facilitate their potential applications.

## Graphical abstract

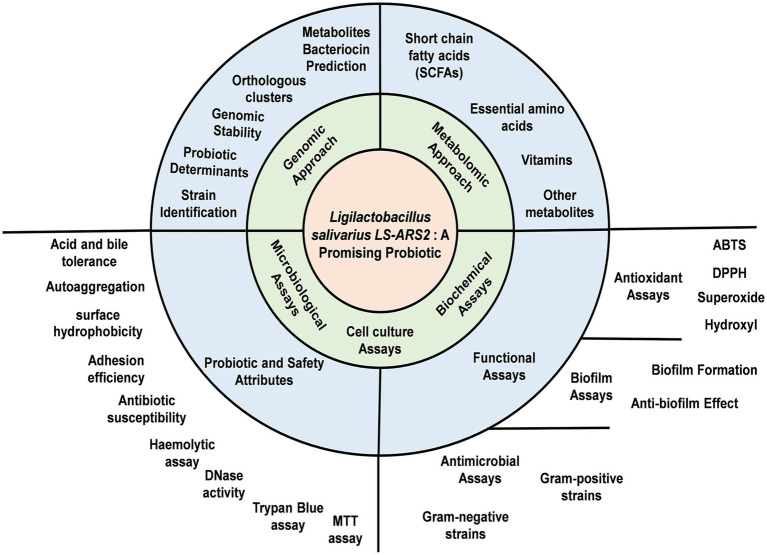

## 1 Introduction

The human gut is colonized by 100 trillion bacteria along with fungi, viruses, protozoans, and archaea, which is collectively called gut-microbiota (Saxami et al., 2023). The gut microbiome is a dynamic ecosystem that manifests a symbiotic relationship with the host. It regulates host metabolism and immunity and preserves intestinal barrier function by restoring a balance between beneficial and pathogenic bacteria in the gut. However, several factors, like diets rich in processed food and refined carbohydrates, unhealthy lifestyles, and uncontrolled medications, jeopardize the equilibrium of gut-microbial composition, which is termed dysbiosis (Pedroza Matute and Iyavoo, 2023). As a result, barrier integrity is disrupted, and permeability is increased, leading to the entry of metabolites and endotoxins from pathogens. This triggers the activation of the immune cells and intestinal inflammation. Constant elevation of inflammatory mediators further promotes chronic inflammatory diseases like cancer, chronic respiratory diseases, atherosclerosis, stroke, and type 2 diabetes mellitus (Hakansson and Molin, 2011). As reported by the World Health Organization (WHO), inflammation-associated diseases are emerging as the most severe health hazard, with around 60% of all deaths across the world (Furman et al., 2019). Interestingly, probiotics can restore microbial balance in the intestine and reduce the severity of gut-inflammation (Patra et al., 2022; Patra et al., 2021). Therefore, probiotic intake as a diet or food supplement could be a preventive strategy for these inflammatory diseases.

Lactic acid bacteria (LAB) are the major source of probiotics. However, not all LAB are probiotics and should be evaluated for their probiotic attributes and safety profile (Ayed et al., 2024). According to the International Scientific Association for Probiotics and Prebiotics (ISAPP), for probiotic application, the strain must be tolerant to harsh conditions (acidic and alkaline) to survive during passage through the gastrointestinal tract. It should have auto-aggregation and adhesion capacity to adhere and colonize the intestinal mucosa. After colonization, the bacteria must have beneficial effects, like the secretion of bioactive metabolites, and/or should depict antimicrobial effects for pathogens. Most importantly, it should be safe for further application (Navarré et al., 2024). Although many potential probiotic strains have been identified, their strain-specific behaviors, safety issues, and lack of reproducibility in animal studies hold their future application (Dash et al., 2023). Therefore, the search for novel probiotics is in constant demand. Moreover, studies on gut-inflammatory diseases have shown that the general physiological benefits of probiotics are strain-specific (McFarland et al., 2018). Therefore, the selection of novel probiotic strains requires well-rounded sequential evaluations for crucial probiotic properties.

Probiotics originating from the intestinal microbiota have a more preventive effect on host health than probiotics isolated from other sources (Timmerman et al., 2006). *Lactobacillus salivarius* is such a LAB that is majorly found in the oropharyngeal-gastrointestinal tract (OGT), milk, vagina, and oral cavities of humans (Yang et al., 2024; Guerrero Sanchez et al., 2022). *Lactobacillus salivarius* was recently reclassified from the *Lactobacillus* genus to a new genus called *Ligilactobacillus* (here onwards, we will follow the same), which refers to its vertebrate host (Guerrero Sanchez et al., 2022). Being a native of the intestinal microbiota, *L. salivarius* helps to maintain a healthy and balanced gut-microbiome with multiple health-benefits (Yang et al., 2024; Zhou et al., 2020; Quilodrán-Vega et al., 2020; Yang et al., 2023; Sevillano et al., 2023; Alba et al., 2025). However, despite their promising potential, compared to other LAB, in-depth studies on *Ligilactobacillus salivarius* strains are scarce (Jiang et al., 2023) (Supplementary Figure S1).

In this above-mentioned background, this study aims to comprehensively characterize the probiotic potential of the strain *LS-ARS2* through whole genome sequencing (WGS) and functional validation. We systematically elucidate the physiological and functional prospects of the strain. For this, we perform genomic, and metabolomic analyses with functional studies to project *LS-ARS2* as a multifaceted probiotic. WGS and phylogenetic analysis confirm the strain as *Ligilactobacillus salivarius.* Genomic predictions and functional assays validate key probiotic traits, including acid and bile tolerance, autoaggregation, cell-surface hydrophobicity, strong adhesion, and robust biofilm-forming capabilities of remarkably fast-growing *LS-ARS2*. The strain also exhibits significant antioxidant properties and potent antimicrobial activity against multi-drug resistant Gram-positive and Gram-negative pathogens, with its cell-free supernatant (CFS) effectively inhibiting pathogenic biofilms. Metabolic profiling through high-resolution mass spectrometry (HRMS) highlights the secretion of diverse health-promoting metabolites. Collectively, this study establishes *LS-ARS2* as a promising probiotic candidate with potential applications as a functional food supplement and biotherapeutic for the improvement of gut-health.

## 2 Materials and methods

### 2.1 Bacterial culture

Pure culture of *LS-ARS2* was procured from Microbial Type Culture Collection (MTCC), Institute of Microbial Technology (IMTECH), Chandigarh, India. The strain was maintained on MRS agar and MRS broth (HiMedia) in anaerobic conditions.

### 2.2 Reaffirmation of the novel strain using whole genome sequencing

#### 2.2.1 Isolation of DNA and preparation of library

*LS-ARS2* was grown in MRS broth anaerobically for 12 h at 37°C. The genomic DNA was isolated using a DNA mini kit [QiaAmp DNA Mini Kit (Cat# 51306)] and quantified by Qubit Fluorometer 3 using the Qubit dsDNA High Sensitivity Assay Kit (Invitrogen, Cat# Q32854). Then, it was further processed for library preparation with the 5,300 Fragment Analyzer (3.1.0.12) using ProSize data analysis software 4.0.0.3. DNA libraries were subjected to pair-end sequencing using the NovaSeq6000 platform (MedGenome, Bangalore, India) with a read length of 151 bp. The generated sequence data was assessed for quality control and processed to generate FASTQ files. The 16S rRNA gene sequencing was performed, and the obtained consensus sequence was analyzed with the NCBI-BLAST tool in comparison with similar sequences present in the repository database. Neighbor-joining (NJ) phylogenetic tree was constructed using MEGA12 to identify the strain (Kumar et al., 2024).

#### 2.2.2 Determination of the strain identity by whole genome sequencing and genome assembly

The whole genome of the strain was sequenced [NovaSeq6000 (MedGenome, Bangalore, India)]. Initially, the reads were refined for contamination with human DNA. According to the alignment to the human genome (around 10.06–27.65%), the reads were filtered for further alignment with a reference genome. The fastq file generated was checked for parameters like distribution of sequence quality score, base quality score, GC content in the reads, average base content per read, over-represented sequences, PCR amplification issue, and adapters. These parameters assisted to ensure the precision of the sequencing results. The sequences of fastq files were trimmed according to the quality report to retain only high-quality sequences for further analysis. Fastq mcf (1.04.803) was used for adapter trimming, a process in which adapter sequences are removed from the 3′ end of the reads. Adapter trimming eliminates the chances of inference (due to adapters) in the alignment of the reads to a reference. For reference-guided assembly, the adapter trimmed reads were mapped to the suggested reference species *Ligilactobacillus sali*var*ius strain* 609_LSAL to get the coverage and assembled fasta. The aligned consensus fasta files were generated [Samtools tools (version 1.2)]. Further, the depth statistics and coverage were generated using bedtools (version 2.0) and an in-house Perl script. The reference-aligned reads were used to predict variants using GATK, and the variants were annotated using the snpEff. The annotation of the primarily assembled genome was performed by applying the Prokaryotic Genome Annotation System (Prokka version 1.14.6) to predict genes, CDS, etc. (Seemann, 2014; Li and Durbin, 2009). The whole genome data can be retrieved from NCBI with the Bioproject Number PRJNA1024881.

### 2.3 Evaluation of probiotic attributes of *LS-ARS2*

#### 2.3.1 Tolerance to acidic pH

An acid tolerance assay was performed, followed by modifications to the previous publication (Abouloifa et al., 2020). *LS-ARS2* overnight culture was inoculated (1% v/v) in MRS broth adjusted to pH 4 and pH 3 with HCl (1 N, Merck), followed by incubation for 0, 1, 3, and 5 h at 37°C (Lab Companion, Korea). MRS broth (pH 6.5) was a control. After each time point, the culture was serially diluted in PBS (pH 7.4), 100 μL culture of appropriate dilution was plated on MRS agar, and incubated under anaerobic conditions at 37°C for 24 h. Colonies were counted, and the viable cells, or the biomass (Log_10_ CFU/mL), were calculated. Survival rate (%) = [biomass in pH 4 or 3/biomass in control pH 6.5] × 100. The experiment was performed in triplicate.

#### 2.3.2 Tolerance to bile salt

A bile tolerance assay was investigated according to the previous publication with modifications (Abouloifa et al., 2020). 1% of overnight culture was inoculated in MRS broth containing 0.3 and 1% (w/v) bile salts (HiMedia). MRS without bile salt was a control. After incubation for 0, 1, 3, and 5 h at 37°C, each culture was serially diluted in PBS (pH 7.4), and 100 μL of the appropriate diluted culture was spread on MRS agar plate, thereafter incubated for 24 h at 37°C under anaerobic conditions. After 24 h, the biomass (Log_10_ CFU/mL) was calculated by counting the colonies. Survival rate (%) = [biomass in bile salt 0.3% or 1% / biomass in control] x 100. The experiment was performed in triplicate.

#### 2.3.3 Determination of self-aggregation property of *LS-ARS2*

The aggregation property was evaluated according to the previous publications with modifications (Fonseca et al., 2021; Campana et al., 2017). 1% overnight culture of *LS-ARS2* was sub-cultured in MRS broth under anaerobic conditions till OD_600_ = 0.5–0.6. The culture was then harvested at 5000 rpm, washed using PBS (pH 7.4) twice, and dissolved in the same (PBS, pH 7.4). The absorbance of the culture was adjusted to approximately 10^8^ CFU/mL (OD_600_ 0.25–0.26 ± 0.1, A_0_) (UV-1800 UV Spectrophotometer, Shimadzu, Japan), vortexed for 10 s, and incubated for 1, 2, 3, 4, 5, 6, 12, and 24 h at 37°C. After each time interval, the absorbance of the upper suspension was measured at 600 nm (A_time_). The auto-aggregation percentage was determined by: Auto-aggregation (%) = [1 – (A_Time_/A_0_) × 100]. *L. acidophilus* DDS1, a widely used probiotic in clinical studies, was used as a positive control. The experiment was performed in triplicate.

#### 2.3.4 Evaluation of cell surface hydrophobicity of *LS-ARS2*

The surface hydrophobicity of *LS-ARS2* was evaluated using the Bacterial Attachment to Hydrocarbons (BATH) method, followed by the previous publication with modifications (Rosenberg et al., 1980). The overnight culture was washed with phosphate urea magnesium sulphate (PUM) buffer and dissolved in 10 mL PUM to reach OD_600_ (A_0_) of 0.8–0.9. The adjusted cell suspension (4.8 mL) and n-hexadecane (Sigma)/xylene (Merck) (0.8 mL) were mixed, followed by incubation for 10 min. The cell suspension was vortexed for 2 min and kept at 37°C for 2 h for the separation of phases. The absorbance of the lower aqueous phase was measured at 600 nm (A). Cell surface hydrophobicity (H%) was calculated as: Hydrophobicity (%) = [(1 – A/A_0_) × 100]. Probiotic strain *L. acidophilus* DDS1 was used as a positive control. The experiment was performed in triplicate.

#### 2.3.5 Determination of adhesion property of *LS-ARS2* using human colon adenocarcinoma cells (Caco-2)

Adhesion ability was studied using Caco-2 cell lines (ATCC), maintained in complete DMEM (Himedia), supplemented with 10% heat-inactivated FBS (Gibco) in 5% CO_2_ (Galaxy 48R, New Brunswick, Germany). 2×10^4^ Caco-2 cells/well were seeded in six-well tissue culture plates (Dash et al., 2023; Ayala et al., 2019). After 80% confluent, the monolayer was washed using PBS (pH 7.4) twice, followed by incubation in serum-free DMEM overnight. Overnight bacteria at MOI (100:1) were co-cultured with Caco-2 cells and incubated for 2 h in a 5% CO_2_ atmosphere at 37°C. Next, cells were washed using PBS (pH 7.4) five times, trypsinized, and gently aspirated. After serial dilution, 100 μL of appropriate dilution was plated on MRS agar followed by incubation for 24 h at 37°C under anaerobic conditions. The attachment efficiency was determined by dividing the average number of bacteria (colonies after 24 h) attached per Caco-2 cell in each well (CFU/cell). The experiment was performed in triplicate.

#### 2.3.6 Elucidation of adhesion properties of *LS-ARS2* using phase-contrast confocal microscopy

To investigate the adhesion property, Caco-2 cells were seeded on poly-L lysine-coated (Sigma) coverslips (HiMedia) in 35 mm plates using complete DMEM and incubated up to 80% confluency (Dash et al., 2023). The cell monolayer was then washed using PBS (pH 7.4), followed by overnight incubation in serum-free DMEM. The bacteria at MOI (100:1) were co-cultured along with Caco-2 cells for 2 h, and unattached bacteria were washed by PBS (pH 7.4). The bacteria attached to Caco-2 cells were fixed with 4% paraformaldehyde for 30 min at 37°C, followed by washing with water. 15 μL of the mounting solution was placed on a glass slide, and the coverslip was placed on the mounting solution upside down. Then, the cells were dried at room temperature, and finally, images were taken in a confocal microscope (FV3000, Olympus, Japan).

#### 2.3.7 Evaluation of biofilm-forming ability of *LS-ARS2*

The biofilm-forming ability of *LS-ARS2* was investigated as described earlier with minor changes (Rocchetti et al., 2024; Baldassarri et al., 2006). Briefly, the *LS-ARS2* strain was grown in MRS broth for 18 h under anaerobic conditions. Then, the culture was diluted in MRS broth till OD_600_ = 0.1 and distributed in 24-well plates. The plates were incubated anaerobically for 24 h at 37°C in a moist chamber. After the incubation period, the media was discarded, and the wells were washed with PBS (pH 7.4), and dried for 1 h at 60°C. The remaining bacterial cells were stained for 45 min with 0.1% (w/v) crystal violet (SRL) solution (dissolved in 95% ethanol), washed with PBS (pH 7.4), and air-dried. The stain was dissolved in 33% acetic acid (Merck), and absorbance was measured at 570 nm. Probiotic strains *L. acidophilus* DDS1 and *L. rhamnosus* GG were used as positive controls. The assay was performed in triplicate.

### 2.4 Determination of safety attributes of *LS-ARS2*

#### 2.4.1 Prediction of prophage, CRISPR sequences, and antibiotic-resistant genes using genome analysis

PHAge Search Tool Enhanced Release (PHASTER) was implemented for the characterization of prophage sequences in the genome of *LS-ARS2* (Arndt et al., 2016). CRISPRCasFinder 1.1.2 (Grissa et al., 2007) was applied to screen CRISPR, Cas sequences, and truncated Cas sequences. The Comprehensive Antibiotic Resistance Database (CARD 3.3.0) (Jia et al., 2016) and the Resistance Gene Identifier Tool (RGI 6.0.3) were employed to find out the genes responsible for antibiotic resistance in the strain sequence using the criteria of perfect and strict hit and high-quality coverage (Zankari et al., 2012). The ResFinder 4.4.2 server was adopted to recognize the genes associated with acquired antimicrobial resistance with a threshold for %ID selected as 90.00% and minimum length selected as 60% and/or chromosomal mutations (Bortolaia et al., 2020). The identification of genes encoding virulence factors and toxins in the *LS-ARS2* genome was performed using a BLAST search against the virulence factor database (VFDB) available at http://www.mgc.ac.cn/cgi-bin/VFs/v5/main.cgi (Liu et al., 2019). The presence of any pathogenic elements in the genome of *LS-ARS2* was examined using the PathogenFinder tool (Cosentino et al., 2013).

#### 2.4.2 Evaluation of antibiotic susceptibility of *LS-ARS2*

The susceptibility of *LS-ARS2* to antibiotics was checked by the disc diffusion pattern following the previous publication (Fonseca et al., 2021). Overnight culture was inoculated on MRS agar plates, and discs containing antibiotics (HiMedia) were placed. Inhibition zone diameters (mm) were measured after incubation for 24 h at 37°C under anaerobic conditions. The susceptibility of the strain to the antibiotics was categorized as resistant (R), intermediate susceptible (I), or susceptible (S) according to CLSI guidelines (CLSI, 2020). The experiment was performed in triplicate.

#### 2.4.3 Assessment of haemolytic activity of *LS-ARS2*

Overnight culture was spot-inoculated on a sheep blood agar plate (defibrinated, 5% w/v, HiMedia) and incubated for 24 h at 37°C (Fonseca et al., 2021). The hydrolysis of blood cells resulted in a clear zone surrounding the colonies, which was considered *β*-hemolysis, whereas green-hued zones surrounding colonies designated partial hydrolysis, and were considered *α*-hemolysis, finally, no zone surrounding colonies was considered *γ*-hemolysis or non-haemolytic. *Staphylococcus aureus* ATCC 25923 was employed as a β-hemolysis positive control, *Escherichia coli* ATCC 25922 as a positive control for α-hemolysis, and reference probiotic strain *L. acidophilus* DDS1 for γ-hemolysis. The experiment was performed in triplicate.

#### 2.4.4 Estimation of DNase activity of *LS-ARS2*

Overnight culture was spot-inoculated on a DNase agar plate (HiMedia) and incubated at 37°C for 24 h (Fonseca et al., 2021). *Staphylococcus aureus* ATCC 25923 was considered a positive control, and the reference probiotic strain *L. acidophilus* DDS1 was a negative control for DNase activity. After incubation, the plates were flooded with 1 N HCl to develop a clear zone around the DNase producer colony. No clear zone around colonies was considered as negative DNase activity. The experiment was performed in triplicate.

#### 2.4.5 Evaluation of safety properties of *LS-ARS2* for human colon adenocarcinoma cells HCT116

The viability of *LS-ARS2*-treated colon cancer cells HCT116 (ATCC) was checked by the trypan blue exclusion assay based on the method described earlier, with modifications (Pradhan et al., 2016). Briefly, 1×10^5^ HCT116 cells/well were seeded in complete DMEM (Himedia) supplemented with 10% heat-inactivated FBS (Gibco), and after attachment, the cells were treated with *LS-ARS2* at 100 MOI and incubated for 6, 12, and 24 h. The media was replenished every 3–4 h to avoid deprivation of the nutrients. After each time point, the cells were harvested and mixed with the same volume of 0.4% trypan blue (HiMedia). HCT116 without *LS-ARS2* treatment was considered a negative control. The living and the dead cells were counted. Cell viability was calculated as the number of viable cells divided by the total number of cells. As a reference, probiotic strain *L. acidophilus* DDS1 was used. The experiment was performed in triplicate.

#### 2.4.6 Assessment of safety properties of *LS-ARS2*-derived CFS in the stationary phase of Caco-2 cells

Cytotoxicity of *LS-ARS2*-derived CFS was assessed on Caco-2 cells according to the previously narrated method with modifications (Pereira et al., 2022). *LS-ARS2* was grown in MRS broth, followed by transfer in complete DMEM for 18 h in a static condition at 37°C. The culture was then centrifuged at 4000 rpm for 20 min at 4°C, followed by filtration through a 0.22 μm filter to collect the CFS. Briefly, 2 × 10^4^ Caco-2 cells/well were seeded in 96-well plates in complete DMEM and allowed to grow for 7 days, with renewal of the media every 48 h. The confluent monolayer of Caco-2 in the stationary phase mimics the normal intestine cells. Hence, the viability of the cells after treatment assessed the safety of *LS-ARS2*-CFS for the host cells (Pereira et al., 2022). On day 7, cells were treated with different concentrations of CFS and pH-neutralized CFS (10, 20, 30, 40, 50, and 60% CFS, diluted with a complete medium) and incubated for 24 h. Caco-2 cells without treatment with CFS were considered a negative control. After the incubation period, 10 μL of MTT reagent (Sigma) was added and incubated for 4 h. Then, 100 μL DMSO (SRL) was added, and MTT absorbance was measured at 570 nm with background correction at 670 nm on the Spectramax iD3. The experiment was performed in duplicate with two technical repeats.

### 2.5 Evaluation of antioxidant properties of *LS-ARS2*

#### 2.5.1 Preparation of the intact and heat-lysed cells

Overnight cultures were washed by PBS (pH 7.4) twice. The cell pellet was re-suspended in distilled water to a final OD_600_ = 1.0. Heat-lysed cells were prepared by heating samples at 95°C (water bath) for 30 min (Ramalho et al., 2019).

#### 2.5.2 Assessment of ABTS cation radical scavenging capacity of *LS-ARS2*

The ABTS cation radical scavenging assay was implemented as stated earlier with minor changes (Kim et al., 2022). A working solution of ABTS (Sigma) was prepared by combining 7 mM ABTS and 2.45 mM potassium persulphate (HiMedia) in equal volume (1:1 v/v). The working solution was diluted with methanol (HiMedia, HPLC) up to OD_734_ = 0.7. 0.6 mL of samples (intact cells or heat-lysed cells) were mixed with 1.2 mL of ABTS solution and incubated for 30 min (dark, room temperature). Distilled water was used as a control. ABTS scavenging rate (%) = (Ac – As)/Ac x 100, where A_C_ is the absorbance of the control and A_S_ is the absorbance of the test sample measured at 734 nm. Probiotic strain *L. acidophilus* DDS1 was considered a positive control. The experiment was performed in triplicate.

#### 2.5.3 Determination of DPPH free radical scavenging capacity of *LS-ARS2*

The DPPH free radical scavenging assay was carried out following the previously described method with minor modifications (Ramalho et al., 2019). 1 mL of sample (intact cells or heat-lysed cells) was mixed with 1 mL 0.2 mM DPPH (CDH) solution in methanol (HiMedia). Mixed vigorously and incubated for 30 min (dark, room temperature). Distilled water was used as a control. DPPH radical scavenging activity (U/mL) = ABS_C_ – ABS_S_/S x 100, where ABS_C_ and ABS_S_ stand for the absorbance of the control and the test samples measured at 517 nm, respectively, and S is the sample volume (mL). Reference probiotic strain *L. acidophilus* DDS1 was considered a positive control. The experiment was performed in triplicate.

#### 2.5.4 Evaluation of superoxide anion scavenging activity of *LS-ARS2*

The superoxide anion scavenging activity was evaluated according to the method described earlier with minor modifications (Gao et al., 2013). 0.8 mL of the sample (intact cells or heat-lysed cells) was added to 0.2 mL of Tris–HCl (Sigma) solution (0.1 M, pH 8). Subsequently, 0.1 mL of pyrogallol (3 mM, Sigma) was added, mixed, and incubated in the dark at room temperature (25°C) for 30 min. The control group was taken as an equal volume of deionized water. Superoxide anion radical scavenging ability (%) = [1 – (A_s_ – A_1_)/A_0_] x 100, where A_s_ = the absorbance (320 nm) of the sample with pyrogallol, A_1_ = the absorbance of the sample solution lacking pyrogallol, and A_0_ = the absorbance of the blank solution with pyrogallol. Reference probiotic strain *L. acidophilus* DDS1 was considered a positive control. The experiment was performed in triplicate.

#### 2.5.5 Estimation of hydroxyl radical scavenging ability of *LS-ARS2*

The hydroxyl radical scavenging assay was evaluated using the earlier method with minor modifications (Xiong et al., 2019). Equal volumes of 2.5 mM 1,10-phenanthroline (Sigma), 0.2 M sodium phosphate buffer (Sigma), and FeSO_4_ (2.5 mM, Merck) were vortexed and incubated for 5–7 min. Then, an equal volume of H_2_O_2_ (0.12% v/v, CDH) and a sample (intact cells or heat-lysed cells) were added, vortexed, and incubated in a water bath at 37°C for 60 min. The control group was taken as an equal volume of deionized water instead of the sample. Hydroxyl radical scavenging activity (%) = [(A_s_ – A_1_)/(A_0_ – A_1_)] × 100, where A_s_ = the absorbance (536 nm) of the sample containing H_2_O_2_, A_1_ = the absorbance of the sample deprived of H_2_O_2_, and A_0_ = the absorbance of the solution with H_2_O_2_ and without a sample. Reference probiotic strain *L. acidophilus* DDS1 was considered a positive control. The experiment was performed in triplicate.

### 2.6 Determination of antimicrobial properties of *LS-ARS2*

#### 2.6.1 Preparation of the cell-free supernatant (CFS)

*LS-ARS2* was grown anaerobically in MRS broth at 37°C for 24 h. The culture was centrifuged at 4000 rpm for 20 min at 4°C. The supernatant was sterilized by filtration through a 0.22 μm PVDF filter (HiMedia) and used for the experiments (Chen et al., 2019).

#### 2.6.2 Evaluation of the antimicrobial activity of *LS-ARS2* using Gram-positive and Gram-negative pathogens, including muti-drug resistant hospital strains

The antimicrobial effect was assessed for enteric pathogens by the agar well diffusion assay using the published method with modifications (Abouloifa et al., 2020). The studied pathogens include enteric Gram-negative strain *Salmonella typhimurium* ATCC 14028, Gram-positive Methicillin-resistant *Staphylococcus aureus* ATCC 700699 (*MRSA*), *Staphylococcus aureus* ATCC 25923, as well as clinically isolated Gram-negative multi-drug-resistant strains like *Escherichia coli* (ETEC) BCH 04067, *Vibrio cholerae* BCH 09616, and *Shigella Flexneri* BCH 06745. The pathogenic strains were swabbed at a concentration of 0.5 McFarland (OD_600_ = 0.5) on Mueller Hinton agar (HiMedia), and 6 mm wells were made on agar medium. The wells were loaded with 100 μL CFS of *LS-ARS2*. The antimicrobial activity was determined by measuring the clear zone of inhibition around the wells in MHA plates after overnight incubation at 37°C. For the negative control, MRS broth (uninoculated) was used. *L. rhamnosus* GG (*LR-GG*), an established probiotic, was used as a positive control. The experiment was performed in triplicate.

#### 2.6.3 Co-culture experiments of *LS-ARS2* with *Salmonella typhimurium*

The antibacterial effect of *LS-ARS2* was further validated by co-culture assay with *Salmonella typhimurium* (ATCC 14028) as described earlier with slight modifications (Dash et al., 2023). Overnight cultures of *LS-ARS2* and *S. typhimurium* were centrifuged and washed with PBS (pH 7.4). First, different doses of *LS-ARS2* (10^6^, 10^7^, and 10^8^ CFU/mL, respectively) were co-cultured with the pathogen (10^6^ CFU/mL) till 24 h, and the viable cells were counted on SS agar plates (HiMedia). To perform the time-kill assay, *S. typhimurium* was co-cultured together with *LS-ARS2* in a 1:1 ratio (10^6^: 10^6^ CFU/mL) for multiple time points (0, 3, 6, 12, 24 h), and respective colonies of *S. typhimurium* (ST) were counted. ST without *LS-ARS2* was considered a control. The experiment was performed in triplicate.

#### 2.6.4 Calculation of minimum inhibitory percentage (MIP) of *LS-ARS2* CFS using broth microdilution method

For the determination of MIP, a microdilution assay was implemented according to the method published earlier (Chen et al., 2019). The overnight culture of *S. typhimurium* was centrifuged and washed by PBS (pH 7.4) twice. CFS was used in three sets: set 1: used as it is; set 2: heated at 95°C for 15 min; set 3: pH of the CFS was neutralized using NaOH. 100 μL of Mueller Hinton broth (MHB, HiMedia) comprising 10^5^ CFU/mL *S. typhimurium* was mixed with different percentages (i.e., 1, 5, 10, 20, 30, 40, 50%) of the CFS diluted in MRS in a microtiter plate. Un-inoculated MHB and MRS were applied as negative controls (blank). The plates were incubated for more than 18 h, and the absorbance was measured at 600 nm using a microplate reader. From the same microtiter plate, cultures were spot-inoculated on MHA plates to evaluate bacteriostatic or bactericidal effects on the pathogen. The standard probiotic *LR-GG* was used as a positive control. The experiment was performed in triplicate.

#### 2.6.5 Evaluation of anti-biofilm properties of *LS-ARS2* CFS

The antibiofilm property of cell-free supernatant of *LS-ARS2* was assessed by the crystal violet staining method according to the previously published method with modifications (Lee et al., 2021). Overnight-grown *MRSA* strain was adjusted to OD_600_ = 0.1 in Tryptone Soya Broth (TSB, HiMedia) and dispensed into each well of 24-well plates with 100 mM glucose (Merck) and NaCl (Merck). CFS of *LS-ARS2* was added to the pathogen at a range of concentrations of 1.25–40% v/v (1/8 MIP to 4 MIP). After incubation at 37°C for 24 h, the medium was removed, and staining was performed as mentioned above (section 2.3.7.). Un-inoculated MRS with TSB broth was used as a control. The biofilm formation rate (%) was calculated according to the following formula: Biofilm formation rate (%) = (OD_Sample_/OD_Control_) x 100. The experiment was performed in triplicate.

#### 2.6.6 Prediction of bacteriocins using the whole genome sequence of *LS-ARS2*

Biosynthetic gene clusters (BGCs) are closely associated groups of genes on a genome that together work to synthesize specialized metabolites. The BGCs of antimicrobial compounds (bacteriocins) of the *LS-ARS2* genome were explored by the web server, BAGEL4 (de Jong et al., 2006).

### 2.7 Determination of functional annotations of the *LS-ARS2* genome

The prediction of genes in the *LS-ARS2* genome was performed using Prokka (1.14.6), and the functional annotation was done using Emapper. The cluster of orthologous groups (COG) intended for the protein-coding genes was determined by Egg-NOG mapper (version 2.1.12) (Dietzsch et al., 2021) using the online Egg-NOG database (version 5.0). The presence of carbohydrate-active enzyme (CAZymes) encoding genes in the *LS-ARS2* genome was explored with the meta server, dbCAN3 (Zheng et al., 2023). Additionally, the genomic determinants linked to various probiotic traits were identified through comprehensive annotation of the *LS-ARS2* genome, employing multiple bioinformatic tools, including KEGG mapper and RAST (Kanehisa et al., 2016; Sethi et al., 2024).

### 2.8 Prediction of primary and secondary metabolites using the whole genome sequence of *LS-ARS2*

The *LS-ARS2* genome sequence was analyzed in gutSMASH (Specialized Primary Metabolite Analysis from Anaerobic Bacteria) for the prediction of potential primary metabolites (Pascal Andreu et al., 2021). Similarly, the secondary metabolites of the strain were predicted using antiSMASH version 6.0.1 (Antibiotic and Secondary Metabolites Shell) using strictness “strict” (Blin et al., 2021).

### 2.9 Elucidation of metabolite profiling of *LS-ARS2* by high-resolution mass spectroscopy (HRMS)

The metabolite composition of the *LS-ARS2*-derived CFS was analyzed using an Exactive™ Plus Orbitrap high-resolution mass spectrometer linked in tandem to an Ultimate 3,000 high-performance liquid chromatography column (Thermo Scientific, United States) (Dash et al., 2023). Liquid chromatography-mass spectrometry (LC–MS)-grade water, methanol, acetonitrile, and formic acid were used (Fisher Scientific, USA). Equal volumes of CFS and methanol (1:1 ratio) were methodically combined and sterilized by syringe filtration using a 0.22 μm filter. For mass spectrometry, around 0.5 mL of the filtered solution was added to the DP ID vial (Cat# C4000-1 W, Thermo Scientific, USA). The components in the sample were sorted out by a C18 column (Hypersil BDS, 250 mm × 2.1 mm, 5 μm; ThermoScientific, USA). The mobile phase contained acetonitrile and water (1:1) containing formic acid (0.1%). The flow rate of the sample was adjusted to 3 μL/min, the temperature of the column was set at 30°C, and the pressure was 700 bar. Electrospray ionization at a potential of 3 eV was used to ionize the molecules. The sample run time was 5 min, and both ions (positive and negative) were detected within the 50–750 m/z scan range. Metabolite annotation of the mass peaks was performed through the National Metabolomics Data Repository (NMDR). The peaks were then annotated with the Metabolomics Workbench database^1^ using public sources such as LIPID MAPS, ChEBI, HMDB, BMRB, PubChem, NP Atlas, and KEGG. For compound consolidation, mass tolerance (the acceptable deviation from the obtained m/z) was set at 0.2 ppm. The results of the analysis were exported with the following filtration criteria: delta PPM ranges from −0.2 ppm to +0.2 ppm and the complete annotated name of the compound. Both positive and negative mode analyses were performed simultaneously, which were combined at a later stage, and duplicates were removed. The analyzed molecules were clarified on the basis of the published literature and recorded in the table.

### 2.10 Statistical analysis

Statistical analysis was performed by two-way ANOVA (for comparison between more than two groups and two parameters) and one-way ANOVA (for comparison between more than two groups and one parameter) to determine significant differences between the treatments using GraphPad Prism (Version 8). The differences were considered statistically significant between all the treatment groups when *p* ≤ 0.05.

## 3 Results

### 3.1 Whole genome sequencing features of *LS-ARS2*

**Figure 1:**
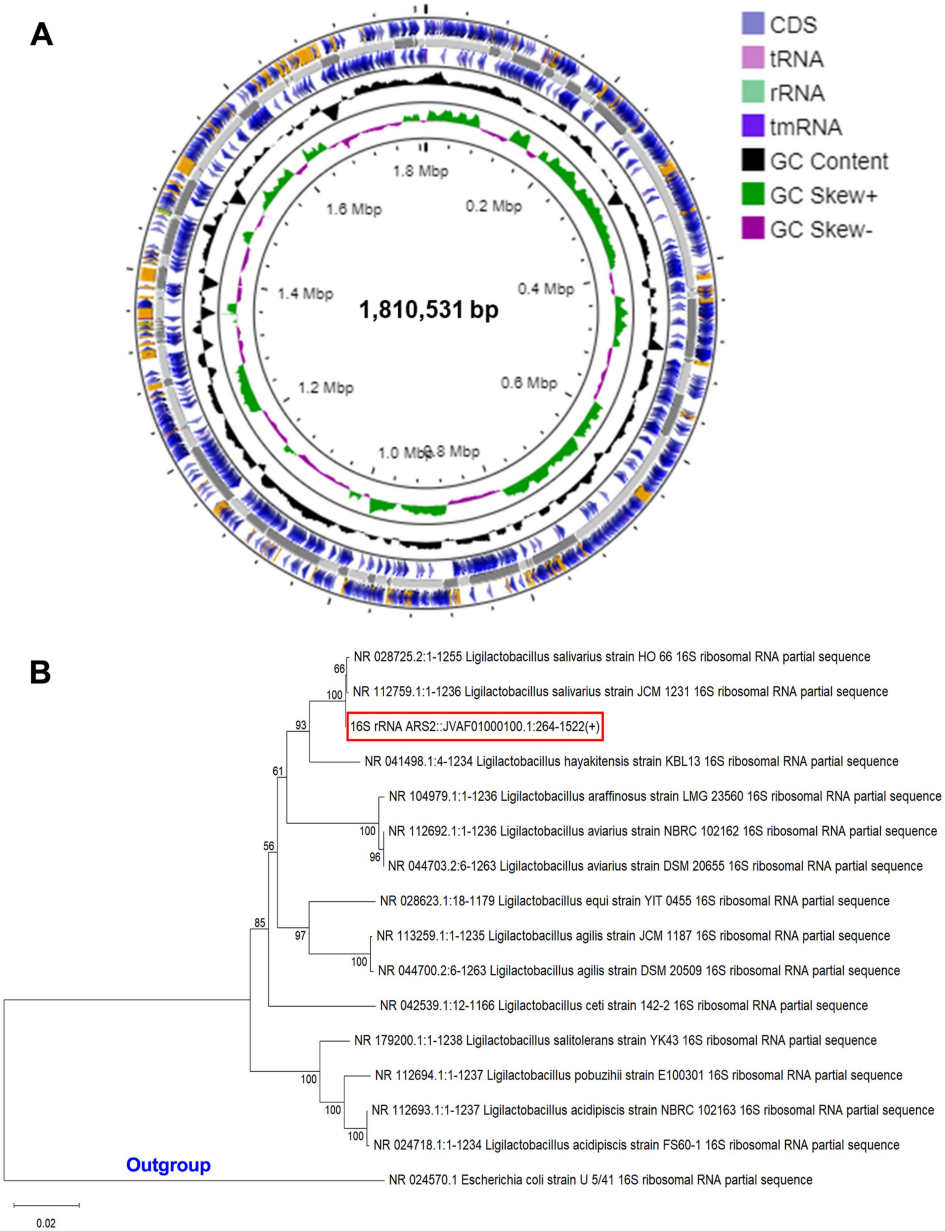
**(A)** Circular genome diagram of *LS-ARS2*. From the inner to the outer: the first ring represented the genome size (1,810,531 bp); the second depicted the GC skew (G + C/G – C); the third and fourth showed forward and reverse CDS (coding sequences) annotated with Prokka (the sites of CDSs/rRNA/tRNA/tmRNA on the genome are marked). **(B)** Phylogenetic analysis based on NCBI blast and MEGA 12 reaffirmed that *LS-ARS2* belongs to *Lactobacillus salivarius.* The phylogenetic tree is constructed based on the neighbor-joining method and indicates that the closest clad for *LS-ARS2* is *Ligilactobacillus salivarius* strain HO 66. *Escherichia coli* U 5/41 is used as an outgroup strain.

Our whole genome sequence study revealed that the genome of *LS-ARS2* was a single circular chromosome. The complete genome sequence contained 1,810,531 bp. Average GC content was found to be 34.4% (Figure 1A). The coding DNA sequences (CDSs) were 1,586, and the identified genes were 1,610 (Table 1). The chromosome contained 4 rRNAs, 19 tRNAs, and 1 tmRNA encoding sequence. The overall summary of the genome assembly was included in Supplementary Table S1.

**Table 1:** Complete genomic feature of *LS-ARS2.*

Strain	*LS-ARS2*
Genome length (bp)	1,810,531
GC content (%)	34.42
Total number of genes	1,610
Coding genes	1,586
tRNA number of the assembled genome	19
rRNA number of the assembled genome	4
tmRNA number of the assembled genome	1
CRISPR-Cas array	3 CRISPR and 5 Cas-associated arrays
Prophage (intact region)	0
Antibiotic acquired genes	NONE

### 3.2 Phylogenetic analysis and taxonomic position reaffirmed the strain as *Ligilactobacillus salivarius*

A BLAST search was performed with all the *Ligilactobacillus* complete genomes to identify the species closest to our strain. Phylogenetic analysis showed the close relations of our strain with other species of the genus *Ligilactobacillus* and grouped in a single clade with *Ligilactobacillus salivarius* strain HO66 (Figure 1B). This phylogenetic analysis result corroborated with the average nucleotide identity (ANI) analysis, where 98% of ANI identity was found for *LS-ARS2* with *L. salivarius* DSM 20555 (Tenea and Ortega, 2021). Moreover, as per 16S rRNA extracted using Barrnapp, *Ligilactobacillus salivarius* strain HO 66 showed the highest identity with our strain. Based on this, our strain was inferred as *Ligilactobacillus salivarius*.

### 3.3 Probiotic attributes of *LS-ARS2:* genomic analysis coupled with *in vitro* investigations

#### 3.3.1 Survival potential of *LS-ARS2*: acid and bile tolerance

Probiotics should be able to survive the harsh environment (temperature, low pH, high bile salts, osmotic pressure, and oxidative stress) of the GI tract to reach the small intestine, where they show beneficial effects.

Interestingly, *LS-ARS2* appears as a fast-growing probiotic. A fast growth rate is not so common among LAB and could facilitate the strain for faster colonization in the gut. Our WGS analysis and functional annotation identified several stress response genes in the *LS-ARS2* genome, like universal stress proteins, *yugI, usp6,* and *uspA,* which are reported to be induced under several stress conditions. *LS-ARS2* genome also harbors numerous heat shock proteins like heat-shock related regulators (*hrcA, ctsR*), molecular chaperons (*dnaK, dnaJ, grpE, groL, groS, hslO, hslU*), and protease encoding genes (*hslU, hslV, clpX, clpL, clpP, clpC, clpE, clpB*) which might protect *LS-ARS2* under heat-shock conditions by preventing intracellular protein aggregation and maintaining membrane stabilization. Additionally, the *cpsC* gene encoding cold-shock proteins was identified in the *LS-ARS2* genome, which might enable the strain to sustain cold stress conditions (Kandasamy et al., 2024).

Being a food supplement, probiotics encounter acidic pH in the stomach after ingestion. Bacteria are reported to evolve direct and specific strategies like ATPases and amino acid decarboxylation-antiporter reactions to remove different stress factors actively. In the present study, functional annotation of the *LS-ARS2* genome identified numerous ATPase-associated genes, which are directly involved in the translocation of protons across the membrane. ATPases are one of the major proton pumps used by Gram-positive bacteria for the extrusion of protons from the cytoplasm by proton motive force. Among them, the most remarkable is F_0_F_1_ ATP synthase, which is an operon consisting of eight genes, *atpA-atpH*, which maintains a stable pH in the bacterial cytoplasm to sustain the acidic stress (Kandasamy et al., 2024). Moreover, several ABC transporters, acyltransferase (*plsC*), pyruvate kinase (*pyk*), alkaline shock proteins (*asp23*), sodium proton antiporter (*nhaC*, which maintains pH and Na^+^ homeostasis), ABC-type Na + efflux pump, and permease component was detected in the *LS-ARS2* genome, which constitutes a stress tolerance machinery helping the strain to survive under acid stress condition (Kandasamy et al., 2024) (Supplementary Table S2).

**Figure 2:**
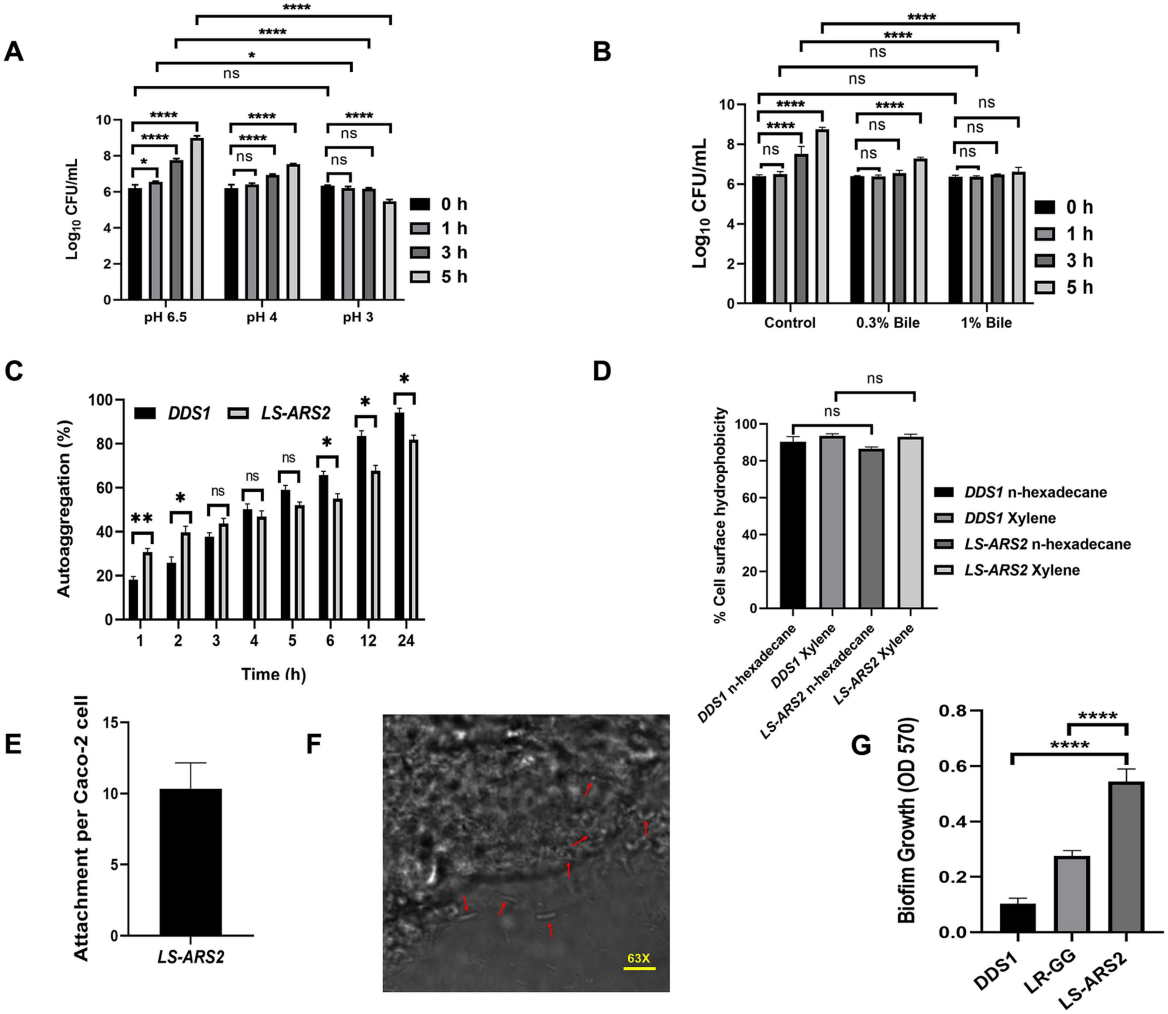
Probiotic properties of *LS-ARS2*. **(A)** Acid tolerance of *LS-ARS2*. Survival of *LS-ARS2* at pH 3 and pH 4 at different time intervals indicated the viability of the strain at acidic pH. Data was presented as mean ± SD from three independent biological replicates. Statistical analysis was done using a two-way ANOVA with a Bonferroni post hoc test; *p < 0.05; ****p < 0.0001. **(B)** Bile tolerance of *LS-ARS2.* Viability of *LS-ARS2* in the presence of 0.3 and 1% bile salt for 3 and 5 h of incubation time indicated tolerance of the strain at high bile concentrations. Data was represented as mean ± SD based on three independent biological replicates. Statistical analysis was done using a two-way ANOVA with a Bonferroni post hoc test; *p < 0.05; ****p < 0.0001. **(C)** Autoaggregation attributes of *LS-ARS2.* The aggregation property of *LS-ARS2* was assessed side by side and compared with the reference probiotic strain *L. acidophilus* DDS1 at different time points up to 24 h. The results were depicted as mean ± SD based on three independent biological replicates. Statistical analysis was done using two-way ANOVA with Bonferroni post hoc test; *p* > 0.05. **(D)** Cell surface hydrophobicity property of *LS-ARS2.* The hydrophobicity percentage of *LS-ARS2* was evaluated with n-hexadecane and xylene (2 h of incubation) in comparison to the reference strain *L. acidophilus* DDS1. All the results were shown as mean ± SD based on three independent biological replicates. Statistical analysis was done using one-way ANOVA with Bonferroni post hoc test; *p < 0.05; ****p < 0.0001. **(E)** Adhesion efficacy of *LS-ARS2*. The attachment efficiency of *LS-ARS2* was shown with intestinal epithelial Caco-2 cells. **(F)** Visualization of Adhesion property of *LS-ARS2.* Phase contrast confocal microscopy images of the Caco-2 cells after co-culturing with *LS-ARS2* showed efficient adhesion to Caco-2 cells. **(G)** Biofilm-forming ability of *LS-ARS2*. The assay was performed compared with two standard probiotic strains, *L. acidophilus* DDS1 and *L. rhamnosus* GG (*LR-GG*). Results were represented as mean ± SD based on three independent biological replicates. Statistical analysis was done using one-way ANOVA with Bonferroni post hoc test; ****p < 0.0001.

Further, the acid tolerance assay showed *LS-ARS2* exhibited 80 and 61% survival in pH 3 after 3 and 5 h of incubation, respectively (Figure 2A). Interestingly, there was no significant difference in the biomass with control MRS up to 1 h of incubation for both pH conditions (*p* > 0.05). Moreover, the viable count increased significantly (****p < 0.0001) after 3 and 5 h of incubation at pH 4. However, in pH 3, the strain survived well up to 3 h (p > 0.05), but the viable cells decreased significantly after 5 h of incubation (****p < 0.0001). Therefore, *LS-ARS2* was able to survive in the acidic pH 3 and pH 4.

Next, the probiotic faces the alkaline conditions of the small intestine due to bile acids. WGS analysis identified inorganic pyrophosphatase (*ppaC*), which maintains membrane integrity and surface tension of bacteria under bile stress conditions. Additionally, several other genes were detected contributing to bile tolerance mechanism of *LS-ARS2,* like cyclopropane-fatty-acyl-phospholipid [CFP] synthase (*cfa*), which enhance lipid synthesis, glutamine synthetase (*glnA*), oligopeptide transporting proteins (*oppA-F*), ABC transporters (*glnR, glnP, glnQ, glnH, glnPH2*), and sodium-bile acid symporter family (Kandasamy et al., 2024) (Supplementary Table S2). Amino acid decarboxylation-antiporter reactions increase the intracellular pH by decarboxylation of amino acid transported into the cell. In the *LS-ARS2* genome, genes encoding Orn/Lys/Arg decarboxylase, C-terminal domain, and possible lysine decarboxylase are annotated, which might be responsible for such mechanism in the removal of stress factors (Dash et al., 2023). Apart from these, *LS-ARS2* contains an abundance of genes involved in carbohydrate metabolism, amino acid metabolism, replication and transcription, and major proteins involved in cell-wall biosynthesis, which reflects the adaptive ability of the strains in various stress conditions (Heunis et al., 2014).

To validate the bile tolerance ability of *LS-ARS2*, we performed a bile tolerance assay. The bile salt concentration varies from 0.2–0.3% depending on the type and amount of food ingested (Hsiung et al., 2021). The strain exhibited 87 and 83% survival in 0.3% (w/v) bile salt after 3 and 5 h of incubation, respectively. The strain also showed a survival of 86 and 76% in 1% (w/v) bile salt after the above-mentioned incubation time (Figure 2B). Interestingly, the survivability of the strain was similar to the control versus 0.3% or 1% bile salt up to 1 h (*p* > 0.05). Moreover, no significant changes were observed in viable count between 0 and 3 h for both 0.3 and 1% bile salt (*p* > 0.05). In addition, the biomass was found to increase significantly (****p < 0.0001) after 5 h in the presence of 0.3% bile. Therefore, the strain could potentially be able to survive at high bile salt.

In summary, our functional annotation and genomic analysis together with the results from biochemical assays strongly supported the functional capabilities and survival of the strain *LS-ARS2* in harsh environments (heat, acid, bile, cold, osmotic) in the GI tract.

#### 3.3.2 Colonization potential of *LS-ARS2:* self-aggregation, cell surface hydrophobicity property, and adhesion efficacy in Caco-2 cells

Probiotics are expected to colonize in the gut and form a strong barrier on the epithelial mucosa that restricts the entry of pathogens.

Functional annotation of the genes by COG analysis exhibited the abundant presence of glycosyltransferases in the *LS-ARS2* genome that might provide a specific benefit to adhere and colonize in the GI tract (Dash et al., 2023). The genomic analysis of the *LS-ARS2* genome identified putative adhesion-related genes such as maltose phosphorylase (*mapA*), lipoprotein signal peptidase II (*lspA*), a sortase family protein (*srtA*), and enolase (*eno*), which are cell-surface proteins responsible for the high adhesion potential of the strain (Kandasamy et al., 2024). The envelope of *Lactobacilli* contains many cell-surface proteins involved in *Lactobacillus*-host interactions. Among them, sortase-dependent proteins (SDPs) and S-layer proteins (SLPs) are best characterized for providing adhesion properties (Lebeer et al., 2008). In the present study, srtA (a sortase family protein) and lspA (involved in adhesion through prolipoproteins) were found by genome COG analysis supporting the adhesion potential of *LS-ARS2*. The genome of *LS-ARS2* also contained several cell wall biosynthetic proteins such as dltD, dltC, dltA, dltB, and dltX, which might influence adhesion capacity and immune response in the host cells (Dash et al., 2023; Lebeer et al., 2008) (Supplementary Table S2).

Since aggregation of microbes is important for gut-adhesion and colonization, evaluation of self-aggregation is considered an important selection criterion for potential probiotics. Our strain showed a significant increase in autoaggregation capacity from 30.6 to 67.6% within 1–12 h, while it showed the highest autoaggregation ability (81.75%) after 24 h (Figure 2C). The auto-aggregation ability shown by the reference probiotic strain *L. acidophilus* DDS1 was also comparable to *LS-ARS2* (^ns^*p* > 0.05, *p < 0.05, **p < 0.05).

Surface hydrophobicity of the bacterial cells is also pivotal for gut-colonization (Hsiung et al., 2021). *LS-ARS2* showed significant surface hydrophobicity with n-hexadecane (86.59%) and xylene (93%) (Figure 2D). The hydrophobicity of *LS-ARS2* was similar to the reference strain *L. acidophilus* DDS1 (*p* > 0.05). This result indicated the potential adhesion capacity of the strain with complex hydrophobic substratum.

One of the most important properties of probiotics is their capacity to adhere to the gut epithelial cells. Therefore, next, we wanted to study directly the adhesion potential of the strain for colon epithelial cells (Caco-2). Significant adhesion capacity (10–12 of *LS-ARS2*/ well-differentiated Caco-2 cells) was observed after 2 h of incubation (Figure 2E). The representative phase-contrast confocal microscopy images also reaffirmed the substantial attachment of *LS-ARS2* to Caco-2 cells. Therefore, the strain indeed showed promising adhesion ability for the human gut lining (Figure 2F).

Therefore, genomic analysis together with *in vitro* assays indicate the potential of the strain *LS-ARS2* to colonize in the gut.

#### 3.3.3 Biofilm forming ability of *LS-ARS2*

Biofilm formation is reported to be a beneficial property of *Lactobacillus* sp. since the formation of biofilm showed better colonization, survival, and prolonged persistence in the gastrointestinal mucosa in the host (Salas-Jara et al., 2016). Probiotic-derived biofilm was also reported to secrete exopolysaccharides, vitamins, enzymes, and many metabolites to enhance the growth of gut-microbiota and inhibit pathogenic adhesion (Gao et al., 2022).

Biofilm-forming ability among *L. salivarius* strains is rare and strain-specific. However, our WGS analysis of *LS-ARS2* revealed the presence of *veg* and *luxS* genes in the *LS-ARS2* genome. It is worth mentioning that *veg* is a widely reported stimulator for biofilm formation in Gram-positive bacteria (Lei et al., 2013). Further, through quorum sensing, *luxS*-derived autoinducer-2 (AI-2) plays a crucial role in the adhesion and formation of biofilm in *Lactobacillus* spp. (Mgomi et al., 2023). Therefore, the presence of the *veg* and *luxS* genes in the *LS-ARS2* genome encouraged us to explore the biofilm-forming ability of *LS-ARS2* experimentally.

It was extremely exciting to find that *LS-ARS2* was able to form robust biofilm within 24 h without any additional carbon source or supplements; such efficacy is rare among *Ligilactobacillus salivarius* strains, indicating the possibility of the strain for efficient and longer sustainability in the gut. Moreover, biofilm formation by *LS-ARS2* was found to be more robust than the reference probiotic strains DDS1 and *LR-GG* (****p < 0.0001) (Figure 2G). *LS-ARS2*-biofilm could, therefore, offer a promising application potential of the strain for improving gut-health.

### 3.4 Safety attributes of *LS-ARS2*: WGS analysis and *in vitro* validation

#### 3.4.1 Genome stability of *LS-ARS2*

Numerous *Lactobacillus* species contain hidden prophages with the potential of cross-contamination or modulation of the intestinal microecology when released (Pei et al., 2021). Therefore, it is essential to perform a comprehensive scanning of prophages in probiotic candidates for their safety evaluation. While analyzing the genome sequence of *LS-ARS2*, we found four prophage regions (PHASTER). However, all of them were incomplete prophage regions (Supplementary Table S3). Moreover, bacteria harbour adaptive antiphage defense mechanisms like the CRISPR/Cas system for their protection from phage attacks (Pei et al., 2021; Roberts et al., 2024). In our strain, three CRISPR arrays and five Cas-associated sequences (CRISPRFinder) were predicted (Supplementary Table S4). Next, a previous study indicated that *Lactobacillus* genomes carrying type I or type III CRISPR-Cas systems harbored fewer intact prophages (Pei et al., 2021). Indeed, TypeIE Cas-associated systems were the most prevalent in the genome of our strain as well. Hence, the presence of a TypeI Cas-associated system in *LS-ARS2* might act as an antiphage defense system and could cause the absence of intact prophage regions.

For the identification of virulence factors (VFs), the genomic sequence of *LS-ARS2* was analyzed against two VF databases: a core dataset comprising experimentally validated VFs and a comprehensive dataset encompassing all known and predicted VF-related genes. While there was no significant hit in the experimentally validated core dataset, analysis against the full dataset revealed the presence of VFG015903 (argK) phaseolotoxin-insensitive ornithine carbamoyltransferase (bits score 56, identities 25%). According to VFDB, this ornithine carbamoyltransferase enzyme is encoded by *Pseudomonas syringae* pv. *phaseolicola* 1448A, which is insensitive to phaseolotoxin-mediated inhibition (Staskawicz et al., 1980). Additionally, the analysis of the pathogenic potential by the PathogenFinder tool classified *LS-ARS2* as a non-human pathogen, with no matches to known pathogenic families and a very low probability (0.159) of being a human pathogen.

Microorganisms may acquire antibiotic-resistant genes by horizontal gene transfer (Jia et al., 2016). Several *Lactobacillus* strains carry d-Ala-d-lactate in their peptidoglycan rather than the d-Ala-d-Ala dipeptide, which makes them resistant to vancomycin (Rychen et al., 2018). For example, *L. plantarum* strains were found with intrinsic vancomycin resistance genes (Deghorain et al., 2007). The genome of *LS-ARS2* was also predicted to have one strict antibiotic-resistant gene (CARD database and RGI analysis, Supplementary Table S5). However, the same was not found with Resfinder analysis.

#### 3.4.2 Antibiotic susceptibility of *LS-ARS2*

Extensive use of antibiotics with food supplements can accumulate genes responsible for antibiotic-resistance in gut-bacteria. These antibiotic-resistant genes can be acquired by the pathogens sharing the corresponding intestinal niche, which appears as a clinical threat (Wong et al., 2015). Therefore, to project any probiotic as a food supplement, the assessment of the antibiotic susceptibility profile is crucial to ensure its safe application. In our study, *LS-ARS2* was sensitive to penicillin, erythromycin, clindamycin, tetracycline, ampicillin, chloramphenicol, and amoxicillin/clavulanic acid. However, it was found to be resistant to vancomycin and gentamicin, similar to many probiotic strains (Table 2). Therefore, *LS-ARS2* was sensitive to the majority of the conventional antibiotics.

**Table 2:** Antibiotic susceptibility of *LS-ARS2* strain determined by the disk diffusion method.

Mode of action	Antibiotics	Results
		*LS-ARS2*
Protein synthesis inhibitors	Erythromycin (15 μg)	S*	Gentamicin (10 μg)	R***	Chloramphenicol (30 μg)	S	Tetracycline (30 μg)	S	Clindamycin (2 μg)	S
	Cell wall synthesis inhibitors	Penicillin (10 μg)	S	Amoxicillin/Clavulanic acid (20/10 μg)	S	Vancomycin (30 μg)	R	Cephalothin (30 μg)	S	Ampicillin (10 μg)	S

Zone diameter (mm) Interpretive Criteria: ≥20 = S* (Susceptible); 15–19 = I** (Intermediate); ≤14 = R*** (Resistant) [CLSI guideline]. The result is presented from three individual biological replicates.

#### 3.4.3 Non-haemolytic and DNase negative properties of *LS-ARS2*

The non-haemolytic nature of *LS-ARS2* was signified by the nonappearance of any transparent zone at the blood agar plate. Further, our strain did not show any halo zone around the colonies at the DNase agar plate, which suggested the absence of DNase activity (Table 3). Therefore, *in silico* studies and *in vitro* assays assured the non-pathogenic nature of *LS-ARS2*.

**Table 3:** Haemolytic and DNase activity of *LS-ARS2.*

Strains	Haemolytic activity			DNase activity
	α	β	γ	
*E. coli* ATCC 25922	+	−	−	NA
*S. aureus* ATCC 25923	−	+	−	+
*L. acidophilus* DDS1	−	−	+	−
*LS-ARS2*	−	−	+	−

*L. acidophilus DDS1, a widely used probiotic in clinical studies, is considered as a reference strain.*

The result is presented from three individual biological replicates.

#### 3.4.4 Safety attributes of *LS-ARS2* in human cell lines

**Figure 3:**
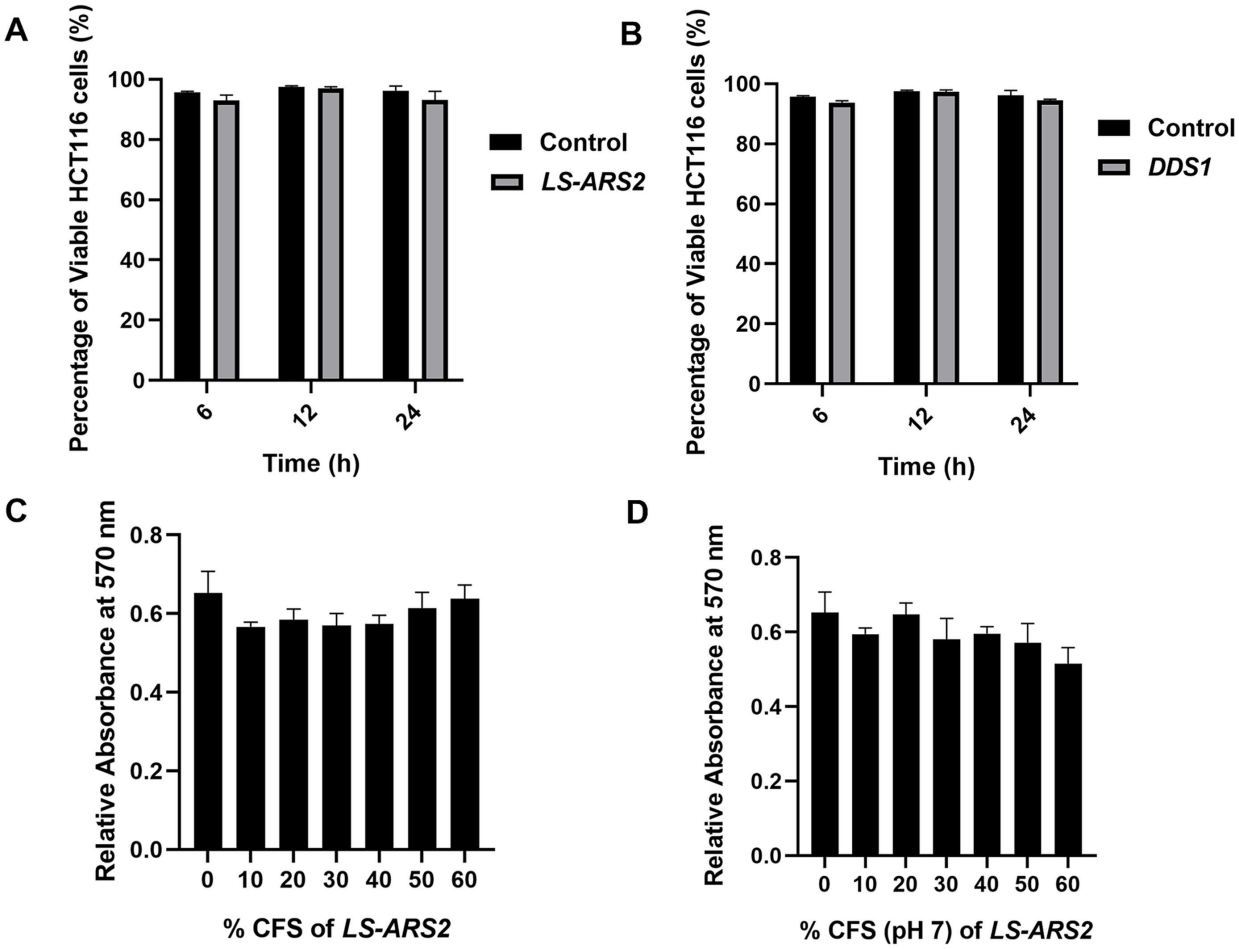
**(A,B)** Trypan blue exclusion assay. The result showed the percentage viability of human colorectal cancer cell HCT116 after treatment with **(A)** *LS-ARS2* and **(B)** *L. acidophilus* DDS1. All the data were represented as the mean ± SD based on three independent biological replicates. **(C,D)** MTT assay. **(C)** *LS-ARS2* CFS and **(D)** pH-neutralized CFS (pH 7) of *LS-ARS2* on Caco-2 cells showed non-toxic nature of *LS-ARS2-*derived CFS.

To further secure the safety attributes of *LS-ARS2*, both the whole cell and cell-free supernatant of the strain were tested on human colon cancer cells. The trypan blue exclusion assay ensured that the application of *LS-ARS2* did not show any notable toxicity to HCT116. A similar result was also observed for the standard probiotic strain *L. acidophilus* DDS1 (Figures 3A,B). In the MTT assay, the CFS of *LS-ARS2* also did not show any notable toxicity as compared to the control (complete DMEM without CFS) to the stationary-phase Caco-2 cells, which mimic the normal intestinal cells (Figures 3C,D). MTT and trypan blue exclusion assays, therefore, ensured the safe application of the strain on human cells.

### 3.5 Functional attributes of *LS-ARS2:* antioxidant potential of *LS-ARS2*

Probiotics with antioxidant potential could alleviate the oxidative stress in the host and, hence, could serve as a natural antioxidant. Functional classification of the *LS-ARS2* genes indicated the presence of three oxidative stress mechanisms: Glutathione: Redox cycle, Glutathione: Biosynthesis and gamma-glutamyl cycle, and Glutaredoxins. Further digging into the *LS-ARS2* genome annotated multiple genes responsible for antioxidant activities (Supplementary Table S2). The genes encoding whole thioredoxin (*trxa, trxb, tpx*) and NADH (*ndh, npr*) antioxidant systems were detected, which are reported to be associated with scavenging of reactive oxygen species (ROS), detoxifying peroxidases, and protecting cells from oxidative stress (Kandasamy et al., 2024; Zhang et al., 2018). Additionally, the genes encoding for glutathione synthetase (*gshF*) and glutaredoxin (*nrdH*) were identified, which are important for the regulation of cellular redox homeostasis (Xiong et al., 2018). Further, genes associated with the methionine sulfoxide reductase system (*msrA* and *msrB*) were recognized in the genome of *LS-ARS2*, which are reported to protect cellular proteins from damage by ROS-mediated oxidation (Oien and Moskovitz, 2019). These genomic signatures encouraged us to find out the potential antioxidant properties of *LS-ARS2* experimentally.

#### 3.5.1 Cation scavenging ability of *LS-ARS2* by ABTS assay

**Figure 4:**
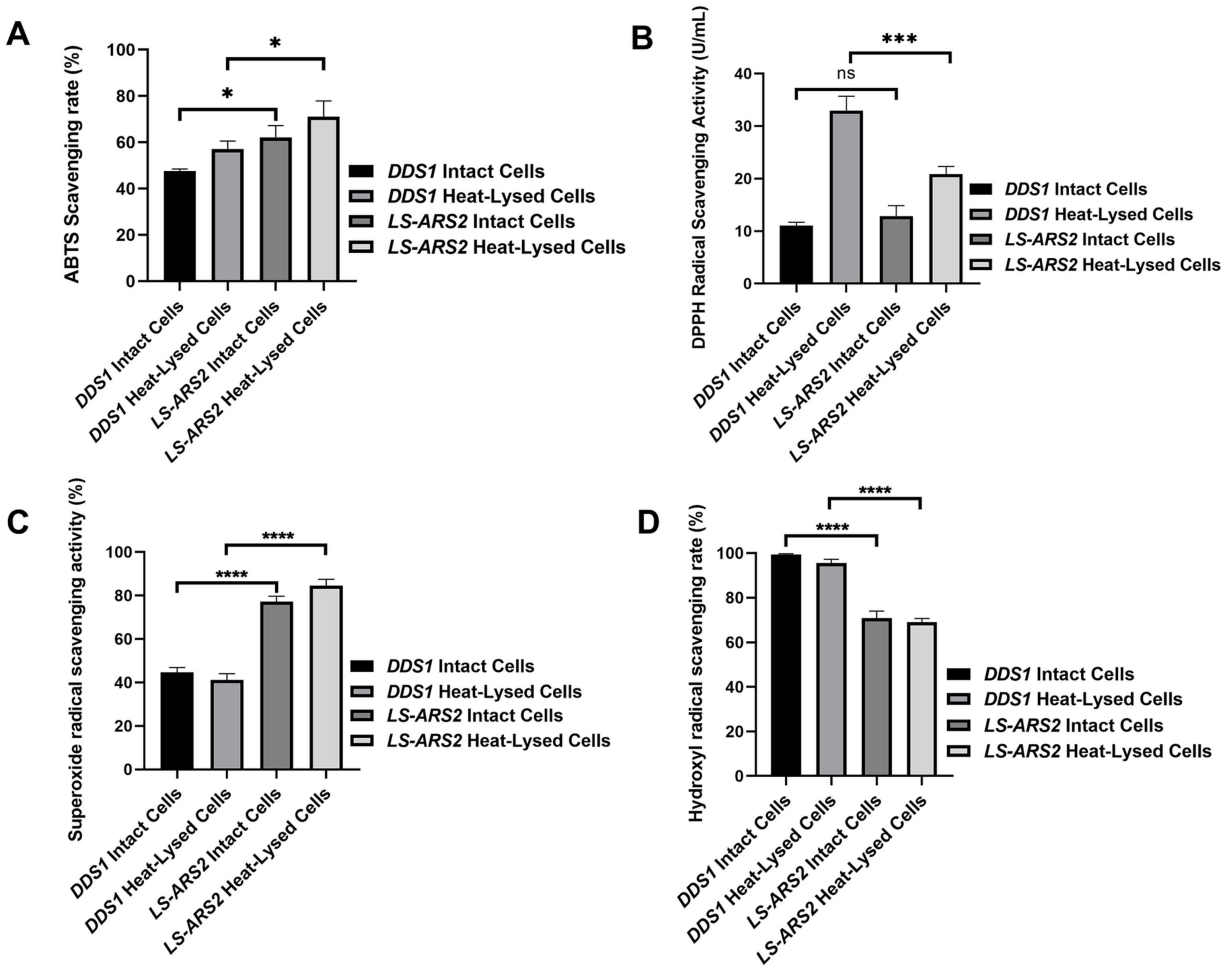
Anti-oxidant properties of *LS-ARS2*. **(A)** ABTS cation radical scavenging assay. The result depicted a significant cation radical scavenging rate of intact and heat-lysed cells of *LS-ARS2*. **(B)** DPPH free radical scavenging assay. The result demonstrated the free radical scavenging activity of *LS-ARS2* intact and heat-lysed cells. **(C)** Superoxide free radical scavenging assay. The data showed the superoxide anion scavenging capacity of *LS-ARS2* intact and heat-lysed cells. **(D)** Hydroxyl free radical scavenging assay. The result indicated hydroxyl radical scavenging ability of *LS-ARS2* intact and heat-lysed cells. All the antioxidant activities were compared with the clinically used probiotic *L. acidophilus* DDS1. The results were shown as mean ± SD from three independent biological replicates. Statistical analysis was done using one-way ANOVA with a Bonferroni post hoc test. *p < 0.05; ****p < 0.0001.

In the ABTS cation radical scavenging assay, the intact cells of *LS-ARS2* showed a 62.14% ABTS scavenging rate, and the heat-lysed cells showed a 71% scavenging rate. Meanwhile, the ABTS scavenging rate of reference strain *L. acidophilus* DDS1 was 47.59% for intact cells and 57.10% for heat-lysed cells (Figure 4A). Therefore, the ABTS scavenging rate of *LS-ARS2* was found to be significant, even higher (*p < 0.05) than the reference probiotic strain *L. acidophilus* DDS1 for both intact and heat-lysed cells.

#### 3.5.2 Free radical scavenging capacity of *LS-ARS2* by DPPH assay

DPPH is used extensively to estimate the free radical scavenging efficacy of antioxidant substances. The transfer of an electron or hydrogen atom to DPPH radical causes a decrease in the absorbance at 517 nm proportionally to the increase of non-radical DPPH (yellow) (Gao et al., 2013). DPPH free radical scavenging activity of *LS-ARS2* whole cell was found to be 12.8 U/mL, which was comparable (*p* > 0.05) to reference probiotic strain *L. acidophilus* DDS1 whole cell (11.08 U/mL). However, the antioxidant activity of *LS-ARS2* heat-lysed cells was found to be significant (20.8 U/mL) but less than the reference probiotic strain *L. acidophilus* DDS1 (32.9 U/mL) (***p < 0.0001) (Figure 4B). It is to be noted that intact bacteria cells show the functional properties of whole cells, whereas heat-lysed form reveals the functionality of intracellular components (Piqué et al., 2019). The intracellular components are strain-specific properties of the probiotics. Therefore, less free-radical scavenging activity of *LS-ARS2* heat-lysed cells than that of DDS1 indicated more significant antioxidant activity of intracellular components of DDS1 than *LS-ARS2*. However, the free radical scavenging capacity of *LS-ARS2* whole cells appeared to be comparable with that of the reference strain, indicating the potential use of *LS-ARS2* as a promising antioxidant agent.

#### 3.5.3 Superoxide radical scavenging ability of *LS-ARS2*

Superoxide anions serve as precursors of singlet oxygen, which indirectly initiates lipid oxidation. The superoxide anion scavenging rate of intact cells of *LS-ARS2* was 77.24%, and heat-lysed cells were 84.61%. Meanwhile, the scavenging rate of reference probiotic strain *L. acidophilus* DDS1 intact cells was 44.68%, and heat-lysed cells were 41.15% (Figure 4C). Hence, *LS-ARS2* depicted remarkable superoxide anion scavenging potency, even higher (****p < 0.0001) than the reference probiotic strain *L. acidophilus* DDS1 for both intact and heat-lysed cells.

#### 3.5.4 Hydroxyl radical scavenging capacity of *LS-ARS2*

Hydrogen peroxide generates hydroxyl radicals in the body as a by-product of various biological processes. This toxic radical initiates indirect oxidation of lipids or proteins and subsequent damage to tissues (Gao et al., 2013). Probiotics may scavenge the radical and reduce such tissue damage. The hydroxyl radical scavenging rate of *LS-ARS2* intact cells was 70.83%, and heat-lysed cells were 69.09%. Reference probiotic strain *L. acidophilus* DDS1 exhibited a 99.41% hydroxyl radical scavenging rate for intact cells and 95.58% for heat-lysed cells. Therefore, the hydroxyl radical scavenging rate of *LS-ARS2* was moderate for both intact cells and heat-lysed cells, although less than the reference probiotic strain *L. acidophilus* DDS1 (****p < 0.0001) (Figure 4D). However, functional properties like hydroxyl radical scavenging capacity are distinct and independent attributes of probiotics. Less activity of *LS-ARS2* than the reference strain could indicate the limitation of the use of *LS-ARS2* as a hydroxyl radical scavenging agent.

### 3.6 Functional attributes of *LS-ARS2:* promising antimicrobial properties

#### 3.6.1 Co-culture assay indicated the significant anti-*Salmonella* effect of the strain *LS-ARS2*

**Figure 5:**
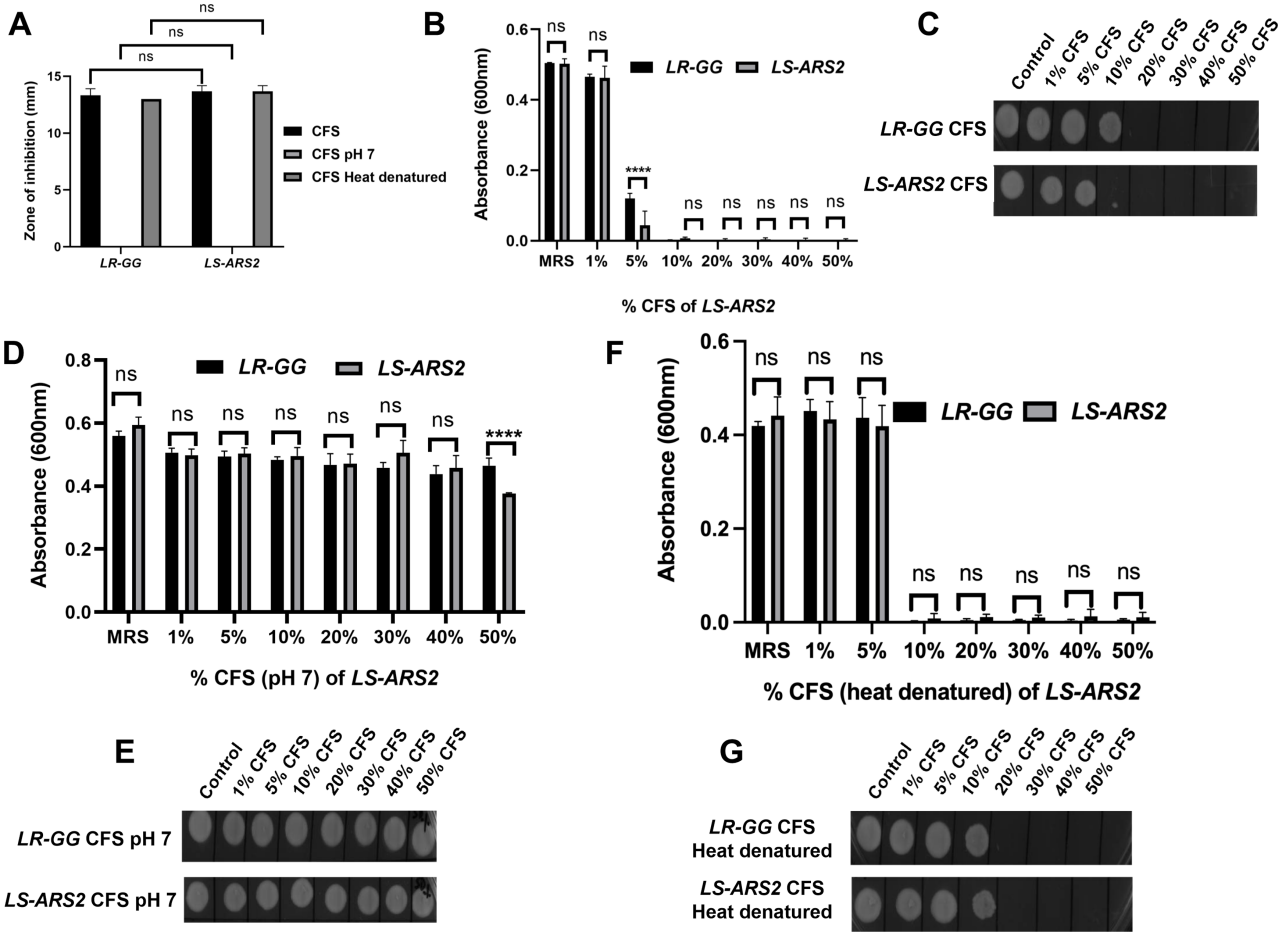
Antimicrobial properties of *LS-ARS2*. **(A)** Agar well diffusion assay. The results showed the effect of normal, pH-neutralized, and heat-denatured cell-free supernatant (CFS) of *LS-ARS2* and *LR-GG* (positive control) on the growth of *S. typhimurium* (ST). The results were shown as mean ± SD based on three independent biological replicates. Statistical analysis was done using two-way ANOVA with Bonferroni post hoc test *p < 0.05; ****p < 0.0001. **(B,D,F)** Determination of the minimum inhibitory percentage (MIP) of *LS-ARS2* CFS for ST. ST was incubated in various percentages of **(B)** normal, **(D)** pH-neutralized, and **(F)** heat-denatured CFS of *LS-ARS2* and *LR-GG* to estimate the MIP of the CFS against the pathogen. The data presented from three biological replicates; *p < 0.05; ****p < 0.0001 using two-way ANOVA with Bonferroni post hoc test. **(C,E,G)** Estimation of the viability of ST in the presence of *LS-ARS2* CFS. From every well of ST grown in *LS-ARS2* CFS and the probiotic *LR-GG* CFS, culture was spotted to find out the bactericidal or bacteriostatic effect **(C,E,G)**. All the data are represented as the mean ± SD from three independent biological replicates.

**Figure 6:**
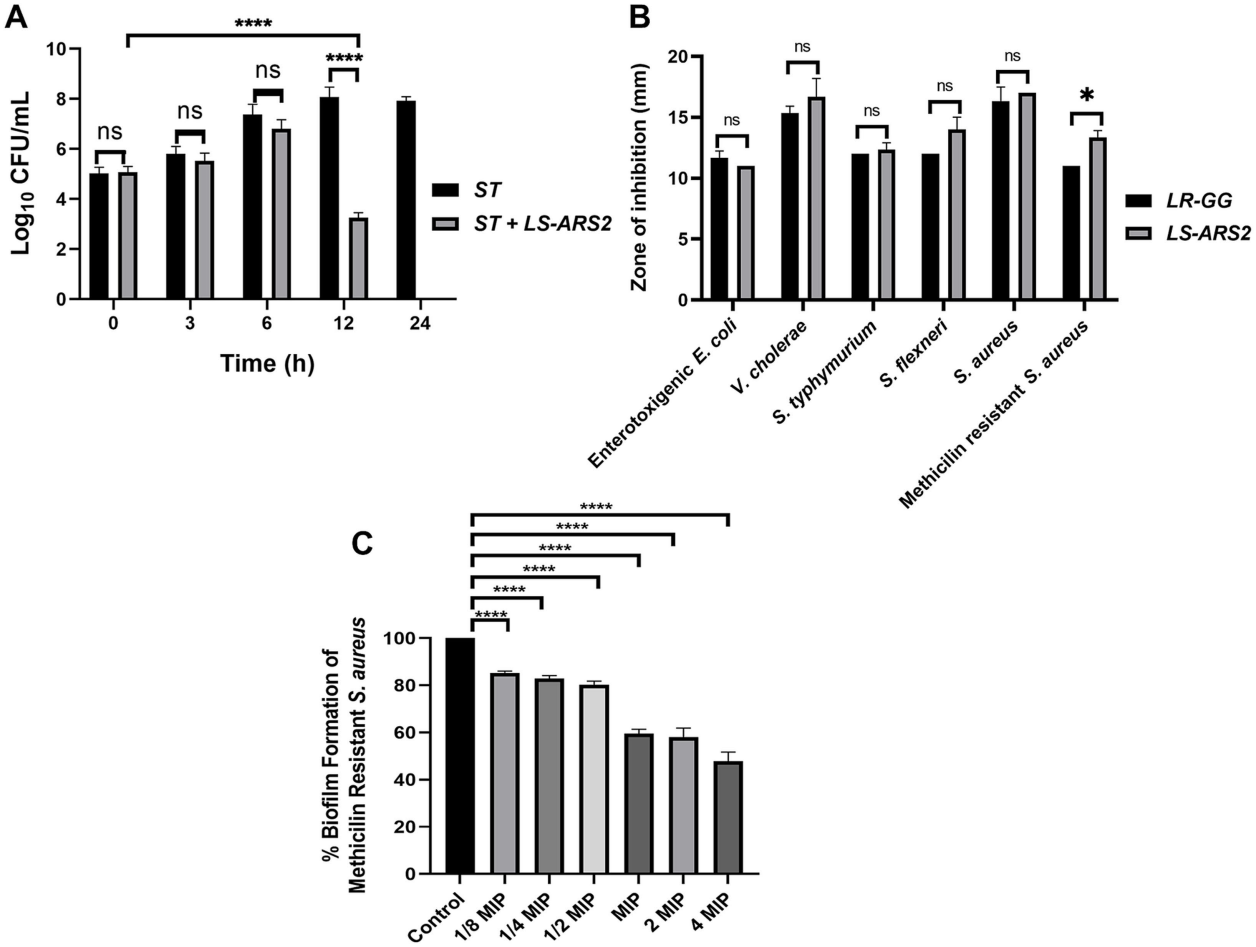
Antimicrobial properties of *LS-ARS2*. **(A)** Co-culture assay. Co-culture of *S. typhimurium* (ST) with *LS-ARS2* at multiple time periods illustrated the *LS-ARS2* cytotoxic effect to the pathogen. After 12 h of co-culture, a significant reduction (****p < 0.0001) in the viable cell number of *S. typhimurium* was observed. Whereas, after 24 h of culture, the viable cell number of ST was decreased to zero (data presented from three biological replicates with multiple technical repeats; *p < 0.05; ****p < 0.0001 using a two-way ANOVA with a Bonferroni post hoc test). **(B)** Agar well diffusion assay. The result showed diverse inhibition zones, signifying the antimicrobial activity of *LS-ARS2* and *LR-GG* (positive control) against multiple Gram-positive and Gram-negative enteric pathogens. The data were represented as mean ± SD based on three independent biological replicates. Statistical analysis was done using two-way ANOVA with Bonferroni post hoc test; *p* > 0.05, *p < 0.05. **(C)** Anti-biofilm assay. Cell-free supernatant (CFS) of *LS-ARS2* showed a significant reduction in the biofilm formation of Methicillin-resistant *Staphylococcus aureus* ATCC 700699. The results were represented as the mean ± SD based on three independent biological replicates. Statistical analysis was done using one-way ANOVA with a Bonferroni post hoc test; ****p < 0.0001.

*Salmonella typhimurium* (ST) is one of the major food pathogens that trigger gut inflammation and life-threatening diarrheal diseases. Therefore, we wanted to evaluate the antimicrobial activity of *LS-ARS2* against *ST*. While characterizing the CFS of *LS-ARS2*, the heat-denatured CFS indicated a zone of inhibition (13.6 mm ± 0.5) similar to the untreated CFS (13.6 mm ± 0.5) in the agar well diffusion assay, whereas the inhibitory effect decreased (0 mm) significantly in the pH-neutralized CFS. This result indicated that the acidic nature of CFS could be the crucial factor for the antibacterial effects of *LS-ARS2* (Figure 5A). This result was similar to the reference probiotic strain *L. rhamnosus* GG (*LR-GG*) (*p* > 0.05). The broth microdilution method showed that 10% of the CFS was enough to inhibit the visible growth of the pathogen and was considered the MIP (Figure 5B). Agar spot assay also suggested that 10% CFS depicted a bacteriostatic effect, whereas 20% CFS was required for a bactericidal effect (Figure 5C). The result shown by *LR-GG* was comparable to *LS-ARS2* (Figures 5B–G). When *LS-ARS2* was co-cultured with *ST*, it effectively hindered the viability of the pathogen with time. A significant (****p < 0.0001) reduction in the viable count of the pathogen was observed after 12 h of co-culture, and complete growth inhibition was observed after 24 h (Figure 6A).

#### 3.6.2 Robust antimicrobial potential of *LS-ARS2* across the diverse pathogen

It is desirable for a potential probiotic to possess robust antimicrobial potential for a wide range of pathogens. Indeed, *LS-ARS2* demonstrated remarkable inhibitory activity against all studied Gram-positive and Gram-negative enteric pathogens (Figure 6B). Among them, the strain showed the highest antimicrobial activity for *S. aureus*, *V. cholerae*, and *S. flexneri*. It should be emphasized that *V. cholerae* and *S. flexneri* were multi-drug-resistant clinical isolates. Moreover, the strain also showed a significant inhibitory effect against Gram-positive methicillin-resistant *S. aureus*. The antimicrobial activity of *LR-GG* was comparable to *LS-ARS2* (Figure 6B) (*p* > 0.05).

#### 3.6.3 Anti-biofilm effect of *LS-ARS2* for methicillin-resistant *Staphylococcus aureus* (*MRSA*)

The pathogenic biofilms pose serious hazards in the food and clinical industries, affecting public health. Therefore, safe applications like probiotics are encouraged to reduce the prevalence of the growth of pathogenic biofilms. *MRSA* is known to be a strong biofilm-former, and *MRSA*-biofilm is a part of their virulence factor (Kaushik et al., 2024).

To estimate the anti-biofilm effect of *LS-ARS2*, first, the MIP of CFS for *MRSA* was determined (10% of *LS-ARS2-*CFS, Supplementary Figure S2). Further, 1/8 MIP to 4 MIP (1.25–40%) of *LS-ARS2*-derived CFS were used to study the formation of *MRSA*-biofilm. Our result indicated a significant reduction (****p < 0.0001) in the formation of *MRSA*-biofilm at all studied concentrations. Moreover, *LS-ARS2*-derived CFS was able to reduce biofilm formation even at 1.25%. These results illustrated the potential of the strain to mitigate the formation of pathogenic biofilm (Figure 6C).

#### 3.6.4 Prediction of bacteriocin in the *LS-ARS2* genome

**Figure 7:**
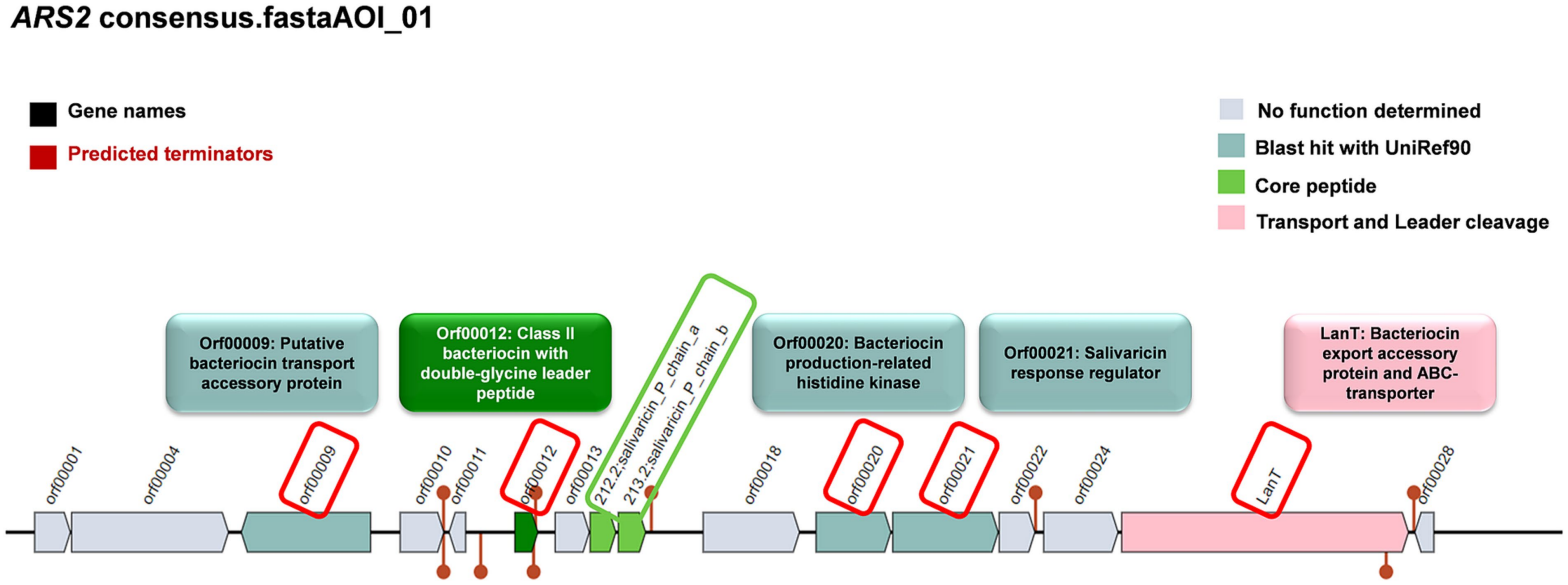
Prediction of genes for bacteriocin and accessory proteins in biosynthetic gene clusters (BCGs) organization in the genome of *LS-ARS2* (BAGEL4 analysis).

The anti-*Salmonella* effect of *LS-ARS2* was eliminated in the CFS, in which the pH was neutralized, indicating the presence of pH-sensitive bacteriocins. This result motivated us to dig further into the *LS-ARS2* genome in search of bacteriocin-encoding gene signatures. It was extremely exciting to find that one area of interest (AOI) region responsible for BCG was identified in the genome of *LS-ARS2* (Figure 7). The BCG was located within contig 32.1 of the salivaricin_P_chain_b class. The AOI of contig 32.1 resides with salivaricin_P_chain_b (bit score = 134.806) and salivaricin_P_chain_a (bit score = 122.479) (Barrett et al., 2007; Elnar et al., 2025). Besides, *LS-ARS2* contains various ORFs encoding miscellaneous bacteriocins (Tenea and Ascanta, 2022), accessory transport, and sensor proteins, as indicated in Figure 7 (Tenea and Ascanta, 2022).

### 3.7 Functional attributes of *LS-ARS2:* prediction from genomic analyses

#### 3.7.1 Association of beneficial metabolite-producing pathways in the *LS-ARS2* genome

**Figure 8:**
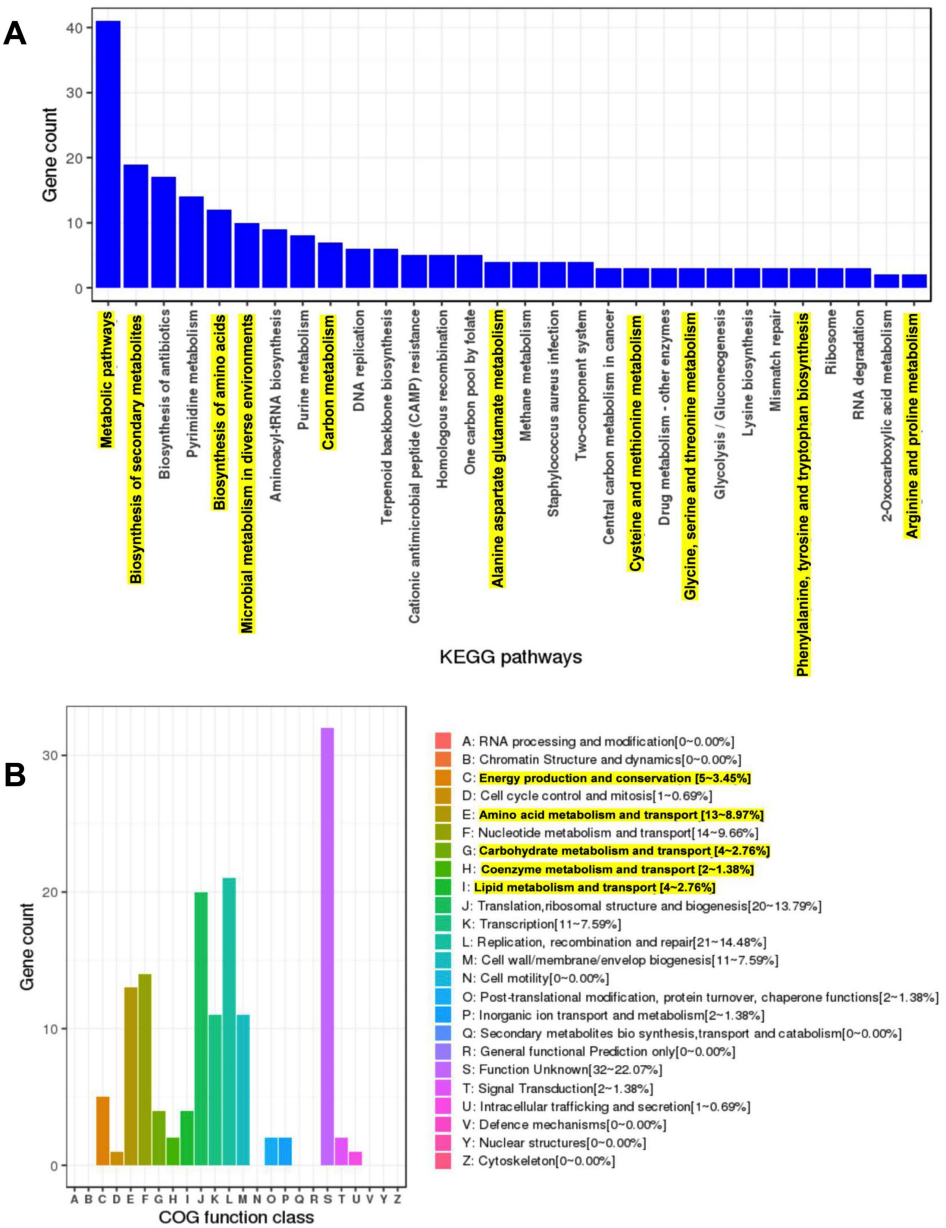
Genome mining of the *LS-ARS2* genome and prediction of major pathways and COG categories. **(A)** The KEGG enriched pathways. Top 30 KEGG enriched pathways were analysed from the genome of *LS-ARS2*. Metabolic pathways, biosynthesis of secondary metabolites, and biosynthesis of antibiotics were three major pathways. Highlighted KEGG pathways showed the gene count of significant metabolite-producing pathways. **(B)** WGS analysis identified major COG categories in the *LS-ARS2* genome. Highlighted COG categories showed the abundant gene count responsible for the production of beneficial metabolites.

The functional potential of *LS-ARS2* was evaluated by annotating its coding sequences using the clusters of orthologous groups (COG) and KEGG databases. The KEGG annotation classified 1,016 CDS (64.1%) into 37 functional classes, 174 pathways, and 30 complete modules (Supplementary Tables S6–S8). Within the annotated KEGG functional categories, the *LS-ARS2* genome showed 30 enriched KEGG pathways. Among them, metabolic pathways contained the highest gene count (approximately 40), followed by the pathway responsible for the biosynthesis of secondary metabolites (approximately 20). Further, the KEGG pathway related to microbial metabolism in diverse environments with an approximate gene count of 10 indicated the adaptation capability of *LS-ARS2* in various niches. Moreover, the strain contained numerous genes involved in major metabolic pathways like amino acid biosynthesis, carbon and nucleotide metabolism, and biosynthesis of cofactors. Interestingly, abundant genes associated with essential amino acid metabolism (methionine, threonine, phenylalanine, and tryptophan) were detected in the *LS-ARS2* genome, which indicated the potential health-benefits of the strain (Figure 8A).

Next, the protein-coding genes of the *LS-ARS2* genome were found to be 1,586, among which 1,527 (96.28%) genes were classified into diverse COG functions. The annotated genes were found to be involved in several housekeeping physiological processes such as replication, recombination and repair (21–14.48%), translation, ribosomal structure and biogenesis (20–13.79%), nucleotide metabolism and transport (14–9.66%), amino acid metabolism and transport (13–8.97%), transcription (11–7.59%), cell wall/ membrane/ envelope biogenesis (11–7.59%), lipid metabolism and transport (4–2.76%), post-translational modification, protein turnover and chaperone functions (2–1.38%), inorganic ion transport and metabolism (2–1.38%), signal transduction (2–1.38%), cell cycle control and mitosis (1–0.69%), intracellular trafficking and secretion (1–0.69%). Functional annotation revealed the association of significant COG categories like energy production and conversion (5–3.45%), amino acid metabolism and transport (13–8.97%), carbohydrate metabolism and transport (4–2.76%), coenzyme metabolism and transport (2–1.38%), and lipid metabolism and transport (4–2.76%) (Figure 8B). Corroborating with the KEGG pathways, the COG analysis also showed the abundance of the genes that could be responsible for the production of beneficial metabolites (amino acids, vitamins) and amino acid or carbohydrate metabolism. The enrichment of metabolic genes in the *LS-ARS2* genome further strengthened the potential of the strain to survive in diverse environments.

In-depth functional classification of the *LS-ARS2* genome indicated the presence of genes involved in the metabolism of key sugars like glucose (*n* = 21), fructose and mannose (*n* = 18), galactose (*n* = 13), starch and sucrose (*n* = 18), amino sugar and nucleotide sugar (*n* = 23), and various other metabolic pathways (Supplementary Table S7). Apart from that, 94 coding sequences were identified for the metabolism of amino acids and derivatives. Among them, 58 were detected to be associated with the metabolism of essential amino acids like valine, isoleucine, leucine (*n* = 6), lysine (*n* = 13), methionine (*n* = 21), phenylalanine (*n* = 3), threonine (*n* = 13), tryptophan (*n* = 2) (Supplementary Table S7). Moreover, the KEGG functional annotation of the *LS-ARS2* genome pointed out 55 coding sequences associated with the metabolism of cofactors and vitamins, including thiamine, riboflavin, nicotinate, vitamin B6, and many more. Such genomic and functional richness strengthens the potential application of *LS-ARS2* as an enriched food supplement. Additionally, comprehensive functional annotation of the *LS-ARS2* genome enabled to detection of the coding sequences associated with carbohydrate fermentation, resulting in the generation of short-chain fatty acids (SCFAs) such as pyruvate (*n* = 22), propanoate (*n* = 10), butanoate (*n* = 7). These carbohydrate metabolic pathways ultimately lead to the synthesis of key SCFAs (acetate, propionate, butyrate, pyruvate, lactate, succinate), characteristic of probiotic activity, underscoring the functional potential of *LS-ARS2*. It is noteworthy to mention here that the presence of genes associated with the metabolism pathways of diverse secondary molecules like terpenoids (*n* = 10) and polyketides (*n* = 4) in the *LS-ARS2* genome emphasized the application of the strain in nutraceuticals and pharmaceutical industries (Supplementary Table S7).

#### 3.7.2 Detection of carbohydrate-active enzymes (CAZymes)

Carbohydrate active enzymes (CAZymes) enable bacteria to adapt to harsh environments by utilizing complex carbohydrate molecules (Liang et al., 2024). KEGG pathways and COG analysis revealed the abundance of genes associated with carbohydrate metabolism and transport, which could enable microbial metabolism in diverse environments. Functional classification of the *LS-ARS2* genome indicated the presence of three pathways involved in carbohydrate fermentation: acetoin, butanediol metabolism, mixed acid fermentation, and lactate fermentation. Further mining into the *LS-ARS2* genome revealed the presence of CAZymes that belonged to the glycoside hydrolase family (GH), glycosyltransferases family (GTs), and carbohydrate-binding modules (CBM) (Table 4). CAZymes of the GH family are responsible for the hydrolysis of complex carbohydrates. In the *LS-ARS2* genome, GH13, GH31, GH77, GH32, GH65, and GH37 were detected, which represents 38.89% of the total CAZyme encoding genes (Supplementary Figure S3). Among these, GH31, GH32, and GH65 are reported to participate in the metabolism of monosaccharides and oligosaccharides such as glucose, galactose, fructose, xylose, or arabinose (Kavanova et al., 2024). Whereas CAZyme families GH13 and GH77 are involved in the hydrolysis of polysaccharides such as *α*-glucans, *β*-glucans, or arabinoxylans. The GH37 family is known to be associated with the degradation of trehalose (Shrestha et al., 2024). In addition, CAZymes involved in the transfer of sugar moieties are termed glycosyltransferases (GTs), which play a key role in forming surface structures sensed by the host immune system (Kandasamy et al., 2024). In the *LS-ARS2* genome, GTs were the most abundantly annotated CAZymes (47.22%), which included GT1, GT2, GT4, GT5, GT26, GT28, GT35, and GT51. Moreover, two types of carbohydrate binding modules (CBM), CBM48 and CBM50, were detected in the *LS-ARS2* genome, representing 13.89% of the CAZymes encoding genes. CBM helps in the binding of some carbohydrate hydrolases to augment the hydrolysis of polysaccharides. The functional annotation also revealed the presence of a few sugar transporter genes (*pts13C, ptsH, ptsI*) and the PTS system of mannose sorbose and fructose family sugars (*manA, manL, manY, manN*) in the *LS-ARS2* genome. Additionally, PTS system genes of fructose family sugars *(dhaK, dhaL, dhaM),* and glucitol or sorbitol group of sugars (*srlA, srlB, srlE, srlM*) were also identified in the *LS-ARS2* genome. Moreover, lactose and cellobiose-specific PTS systems like *mtlA, mtlD, mtlF, and mtlR* were identified in the genome of *LS-ARS2*. The presence of PTS system encoding genes indicated the strong carbon transporting ability of *LS-ARS2*, which is crucial to adapting multiple habitats (Kandasamy et al., 2024). Fascinatingly, genes encoding alcohol dehydrogenase, phosphoketolase, phosphate acetyltransferase, and pyruvate formate-lyase were identified in the *LS-ARS2* genome, which is important for homo- or hetero-fermentative pathways making *LS-ARS2* an attractive choice in the food industry (Kandasamy et al., 2024) (Supplementary Table S2).

**Table 4:** Carbohydrate active enzymes (CAZymes) detected in the *LS-ARS2* genome.

Preferred name	Cazyme	Function
pbp2A	GT51	Penicillin-binding protein
tagA	GT26	Participates in the *de novo* synthesis of teichoic acid
xlyB	CBM50	LysM domain
murG	GT28	Cell wall formation
mgs	GT4	Glycosyltransferase, group 1 family protein
cpoA	GT4	Glycosyltransferase, group 1 family protein
yfdH	GT2	Glycosyltransferase, group 2 family protein
treC	GH13	Alpha amylase, catalytic domain protein
ponA	GT51	Penicillin-binding protein 1A
ascB	GT1	Belongs to the glycosyl hydrolase 1 family
malQ	CBM48, GH13, GH31, GH77	Belongs to the glycosyl hydrolase 13 family
glgP	GT35	Phosphorylase is an important allosteric enzyme in carbohydrate metabolism
glgA	GT5	Synthesizes alpha-1,4-glucan chains using ADP-glucose
glgD	GT5	Nucleotidyl transferase
glgB	CBM48, GH13, GH31	Catalyzes the formation of the alpha-1,6-glucosidic linkages in glycogen
nplT	GH13	Belongs to the glycosyl hydrolase 13 family
recX	GT4	Regulatory protein RecX
waaB	GT4	Glycosyl transferases group 1
mltD	CBM50	NlpC P60 family protein
nplT	GH13	Belongs to the glycosyl hydrolase 13 family
scrA	GH32	Invertase
mapA	GH65	Hydrolase, family 65, central catalytic
pgmB	GH37, GH65	Beta-phosphoglucomutase
mltD	CBM50	Lysin motif

### 3.8 Metabolomics of *LS-ARS2:* genomic prediction coupled with experimental validation by HRMS analysis

#### 3.8.1 Prediction of primary and secondary metabolites in the *LS-ARS2* genome

**Figure 9:**
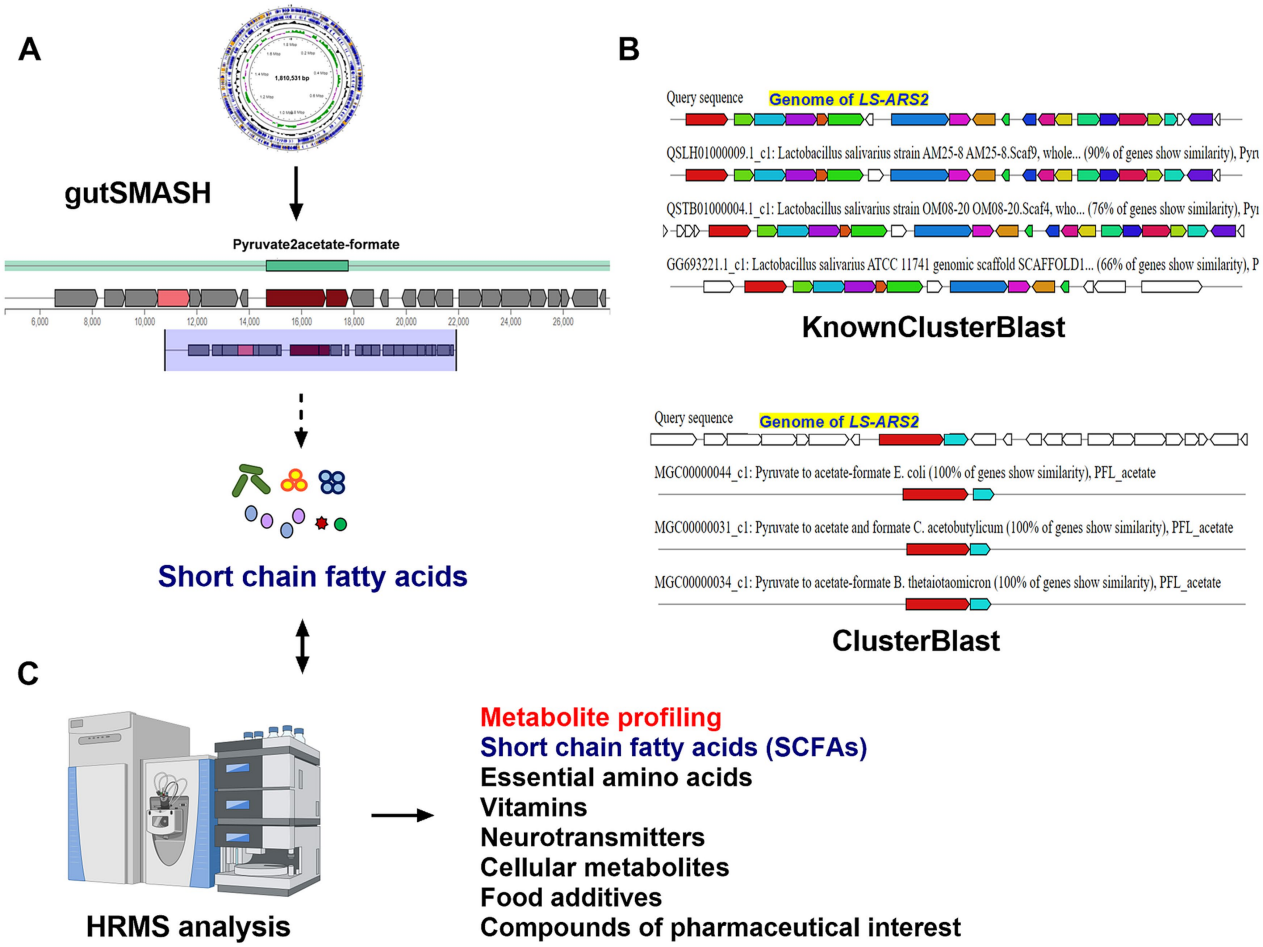
Prediction of genes for primary metabolites in Metabolic Gene Clusters (MGCs) of the *LS-ARS2* genome. **(A)** GutSMASH run of the *LS-ARS2* genome. GutSMASH predicted one MGC region, type pyruvate 2 acetate-formate, which is responsible for the production of SCFAs. **(B)** Gene Cluster Comparative Analysis. Comparative analysis between different bacterial reference genes (pre-computed in gutSMASH) for the most similar metabolic gene clusters (MGCs) based on a gutSMASH run. Genes with the same colour indicated putative homologs based on significant Blast hits between *LS-ARS2* and the reference bacterial genes. The KnownClusterBlast analysis showed the gene similarity of the predicted region (Pyruvate2acetate-formate) with MGCs associated with known functions of other *Lactobacillus* reference genomes. The ClusterBlast analysis output showed that the predicted metabolic gene cluster (MGC) (Pyruvate2acetate-formate) does not have homologous MGCs among other *Lactobacillus*. White genes have no relationship. **(C)** Detection of beneficial metabolites in the *LS-ARS2* CFS using HRMS analysis (also see Table 5 for details).

Microorganisms produce various substances (primary and secondary metabolites) that modulate host metabolism and immunity. Metabolic gene cluster (MGC) is defined as a cluster of genes that encodes different enzymes of the same metabolic pathway. The gutSMASH tool predicts genes involved in bioenergetics and metabolism in anaerobic bacteria (Pascal Andreu et al., 2021). In our study, gutSMASH found one MGC region, pyruvate to acetate-formate type (region 58.1), in the *LS-ARS2* genome (Figure 9A). This region encodes for short-chain fatty acids (SCFAs), primarily acetate, butyrate, and propionate, which could have a positive impact on gut-health (Donia and Fischbach, 2015). The KnownClusterBlast output indicated high sequence similarity between the predicted *LS-ARS2* MGC and other *Lactobacillus* strains (Figure 9B). Interestingly, among all the *Lactobacillus* strains, the highest gene similarity of the Pyruvate2acetate-formate region was found with other *L. salivarius* strains with the same region and known function. The KnownClusterBlast output thus strongly supports the potential production of primary metabolites (SCFAs) from *LS-ARS2*. The gutSMASH results nicely corroborate with the previous KEGG pathways and COG analysis, where abundant gene count was detected for metabolic pathways (specifically carbohydrate metabolism and transport) and energy production. The output of the gutSMASH further correlated with the functional prediction where several genes, like *nagA, pdh, fabI, acc, poxB, gabD,* and *fadB4,* associated with the SCFA biosynthesis were identified in the genome of *LS-ARS2*.

The KEGG pathway analysis indicated the richness of genes responsible for the biosynthesis of secondary metabolites, which are vital for the antibacterial, antifungal, and antitumor activities of the microorganisms (Tenea and Ascanta, 2022). The web server, antiSMASH, predicts NRPs, PKs, terpenes, and RiPP-like peptides with various known or putative secondary metabolites in the genome of bacteria. Fascinatingly, further genome mining of the *LS-ARS2* genome by antiSMASH predicted one secondary metabolite-producing region (region 39.1) named T3PKS region (T3PKS: Chal_sti_synt_N, Chalcone and Stilbene synthases, N-terminal domain) within the *LS-ARS2* genome (Supplementary Table S9). Polyketide synthases (PKSs) are related to the synthesis of antibiotics and pharmaceutical products and have various roles in food processing (Naughton et al., 2017). Microbial Type III PKSs (T3PKS) are involved in the biosynthesis of some lipidic compounds and various secondary metabolites with significant biological functions (Katsuyama and Ohnishi, 2012). Notably, the results from antiSMASH analysis aligned with genomic predictions from the KEGG pathways analysis, particularly those associated with the metabolism of terpenoids and polyketides (Supplementary Table S6).

#### 3.8.2 Beneficial metabolites in *LS-ARS2* CFS: HRMS analyses

The detailed genome mining and functional annotation of the *LS-ARS2* genome showed the abundance of the genes responsible for synthesizing SCFAs, vitamins, essential amino acids, and cellular metabolites (Figures 8A,B, 9A,B; Supplementary Tables S2, S7). Next, we explored metabolic profiling of the strain to integrate WGS-based functional predictions with the HRMS-generated metabolites. The whole genome mining by gutSMASH tool identified the major SCFA-producing MGC, pyruvate2acetate-formate, in the *LS-ARS2* genome. Further, the thorough investigation of the KEGG pathways and functional annotations of the *LS-ARS2* genome predicted the gene signatures like *acyP, pdhC, accC, accD, accB, fabD, pykfadB4,* and *argE*, associated with the SCFAs biosynthesis (Supplementary Table S2). Notably, the HRMS analysis indicated the presence of a wide range of organic acids (for example, lactic acid, acetic acid, butyric acid, and homoserine) in the CFS of *LS-ARS2,* correlating metabolic pathways and gene signatures predicted in the genome. Several SCFAs, like butyric acid, isobutyric acid, lactic acid, acetic acid, and hydroxypropionic acid, were also prominent in CFS, aligning with the genomic identification of genes involved in SCFA biosynthesis (Figure 9C). These compounds have been shown to function as major antimicrobial components and immunomodulators (Dash et al., 2023). The detection of acetic acid, hydroxypropionate, butyric acid, and lactic acid in the metabolic profile strongly correlated with the genes encoding enzymes linked to glycolysis, pyruvate, propanoate, butanoate metabolism, and fermentation pathways. Additionally, the presence of many essential amino acids like leucine, isoleucine, valine, phenylalanine, threonine, and lysine in the CFS of *LS-ARS2* was consistent with the genomic prediction of KEGG pathways involved in amino acid metabolism (Figure 8A; Supplementary Table S7) (see discussion). The WGS-genome mining identified KEGG pathways associated with the metabolism of vitamin B6 and nicotinate and nicotinamide (Supplementary Table S7). The functional annotation further underscored the gene signatures, including *pdxK, nadD, birA, folA, thiT, adk,* which are involved in vitamin metabolism. The identification of vitamins such as pyridoxal (vitamin B_6_) and nicotinic acid (vitamin B_3_) by metabolomic analysis in the *LS-ARS2*-derived CFS provided a robust correlation between the predicted metabolic pathways and gene signatures with the HRMS metabolite profiling (Table 5; Figure 9C). Therefore, our integrated genome mining and HRMS analysis beautifully validated the findings from the WGS analysis and confirmed the presence of beneficial metabolites in the CFS of *LS-ARS2,* reinforcing the functional capabilities of the strain.

**Table 5:** Metabolite profiling of the cell-free supernatant (CFS) of *LS-ARS2.*

Name	Input mass	Matched mass	Significance
Butyric acid	89.02	89.0597	Energy source for colon cells, important for gut-health, reduction of inflammation
Phenylalanine	166.1	166.0862	Essential amino acid
Acetate	60.04	60.0206	Fermentation and acidification, cholesterol synthesis
Cysteine	122.5	122.0270	Protein synthesis and other metabolic functions
Betaine	116.1	116.0717	Lactic acid fermentation, prevention of liver injury
Threonine	118.1	118.0510	Protein synthesis
Gamma-aminobutyric acid	102	102.0561	Neurotransmitter
Fructose	179	179.0561	Cellular metabolite
Glucose	179	179.0561	Cellular metabolite
Citric acid	191	191.0197	Mineral absorption, source of flavonoids, antioxidants, and vitamin C
Dihydroxyacetone	89.02	89.0244	Used in the cosmetic industry, intermediate in lipid biosynthesis, and glycolysis
Lactic acid	89.02	89.0244	Antimicrobial, antiviral and immunomodulatory properties
Dimethylglycine	102	102.0561	Important for boosting energy and immunity
Nicotinic acid	122	122.0248	Important for the treatment of dyslipidemia
Arabinonic acid	167	167.0550	Takes part in an organism’s growth, development, or reproduction
Leucine	132.1	132.1019	Essential amino acid
Isoleucine	132.1	132.1019	Essential amino acid
Pyruvic acid	89.02	89.0233	Potential anti-inflammatory and antioxidant agent
Acetoin	89.02	89.0597	Flavour additive in food, cosmetics, synthesis of optically active pharmaceuticals
Valine	118.1	118.0862	Essential amino acid
Oxaloacetate ion	132.1	132.0053	Cellular metabolite. Can alleviate liver injury
Isobutyric acid	89.02	89.0597	Energy source for colonocytes and has antimutagenic activity
Methyl propionate	89.02	89.0597	Antibacterial and antifungal activity
3-Hydroxypropionate	89.02	89.0233	Useful as a platform for synthesis of biodegradable plastic
Acetylglycine	118.1	118.0499	Has a role as a metabolite in peptide and amino acid modification
Aminocaproic acid	132.1	132.1019	Useful in the treatment of reducing bleeding and cardiac surgery
Oxoglutaric acid	145	145.0143	Cellular metabolite
Methylglutaric acid	145	145.0506	Organic acid can reduce cholesterol synthesis
Glutamine	145	145.0619	Protein synthesis
Lysine	145	145.0983	Can reduce anxiety, and cold sores, improve calcium absorption, protein synthesis
Acetylcholine	145	145.1108	Major neurotransmitter
Homoserine Lactone	102	102.0549	Quorum-sensing
Aspartic acid	132.1	132.0302	Protein synthesis
Homoserine	118.1	118.0146	Antibacterial and anticancer activity
2.3-butanediol	89.02	89.0608	Has applications in chemical, cosmetics, agriculture, and pharmaceutical industries
Pyridoxal (vitamin B_6_)	166.1	166.0510	Metabolism of lipids, carbohydrates, coenzymes, and hormones. Brain function
Malonic semialdehyde	89.02	89.0233	Important for cosmetic and pharmaceutical industries
Adipic acid	145	145.0506	Has applications in food and beverages, pharmaceuticals
5-keto-D-fructose	179	179.0550	Natural diketone, present in honey
Gamma-amino-beta-hydroxybutyric acid	118.1	118.0510	A derivative of the neurotransmitter gamma-aminobutyric acid
Ethanolamine	60.04	60.0455	Diverse application in pharmaceutical and chemical industry
3-Aminobutanoic acid	102	102.0561	Secondary metabolite, can act as a defense or signaling molecule

## 4 Discussion

Despite their promising potential as probiotics, very few *L. salivarius* strains are available at an industrial scale as commercial products (Guerrero Sanchez et al., 2022). Our results illustrate that *LS-ARS2* is a biofilm-former LAB, that exhibits significant antioxidant, antibacterial, as well as antibiofilm potential. Metabolic profiling of *LS-ARS2*-CFS indicates the presence of diverse health-promoting metabolites as well as other compounds with various applications as food, poultry, and cosmetic products. Therefore, *LS-ARS2* appears as a promising candidate for diverse lucrative applications, particularly in food and therapeutic industries (see below for details).

First, the WGS analysis, together with the *in vitro* assays, establishes the probiotic attributes of *LS-ARS2.* The strain could potentially withstand the acidic and alkaline environments of the stomach and small intestine, respectively. Biofilm formation ability, aggregation efficacy, as well as adhesion and colonization potential, could facilitate *LS-ARS2* for an efficient and longer stay in the gut. Moreover, the safety attributes of the strain are also assured, although at the *in vitro* level.

The dysbiotic gut often disrupts the intricate oxidative balance (McCord, 1985). Supraphysiological levels of all the reactive oxygen species (ROS), like hydrogen peroxide and superoxide anions, cause severe tissue damage and organ dysfunction (Barry, 1991). The excess level of ROS triggers chronic inflammation that results in gut-inflammatory diseases (ulcerative colitis, intestinal bowel syndrome, colorectal cancer) as well as diseases related to gut-organ axes (Ren et al., 2023). The significant antioxidant activities of *LS-ARS2* reveal the potential of the strain to reduce oxidative stress in the gut. Interestingly, the functional annotation of the *LS-ARS2* genome beautifully corroborates our *in vitro* investigations (Figure 4), indicating that supplementation of *LS-ARS2* could contribute to the oxidative balance in the gut.

Chronic infections by pathogenic biofilms are clinically challenging. *MRSA*, particularly in biofilm form, is a leading cause of hospital-acquired infections that cause significant morbidity and mortality. *MRSA*-biofilm facilitates the pathogen to invade, spread, and resist antimicrobial treatments (Ciofu et al., 2022). In this background, it is extremely exciting to find that the CFS of *LS-ARS2* inhibits *MRSA*-biofilm. Application of *LS-ARS2* could therefore provide a green therapeutic window for combating pathogenic-biofilm.

WGS analysis predicts the presence of the MGC region (encodes SCFAs) in the genome of *LS-ARS2*. The HRMS study indicates the presence of diverse SCFAs (acetate, propionate, butyrate, lactic acid, etc.) in the CFS of *LS-ARS2*. Thus, genomic characterization coupled with metabolomic studies (Figure 9) confirms that, like other probiotic bacteria, SCFAs in the CFS of *LS-ARS2* could offer beneficial roles to the host-gut. These SCFAs eliminate pathogens by entering through their cell membrane and acidification of their alkaline cytoplasm. Further, SCFAs have numerous health-promoting properties, including antibacterial, antiviral, immunomodulatory, and anticancer effects (Dash et al., 2023). Specifically, butyrate is an energy source for colonocytes (Pedroza Matute and Iyavoo, 2023). Therefore, intake of *LS-ARS2* could facilitate maintaining gut-health.

Bacteriocins, produced by LAB, are ribosomally synthesized antimicrobial peptides. BAGEL4 analysis of the *LS-ARS2* genome shows the presence of one class II bacteriocin-producing BCG (salivaricin_P_chain_b) and bacteriocin accessory as well as export proteins, which supports the probable presence of bacteriocin in the *LS-ARS2* genome. Bacteriocins and associated proteins retain their activity preferably at an acidic pH rather than at an alkaline or neutral pH (Magnusson and Schnürer, 2001). In the present study, the antimicrobial effect of the *LS-ARS2* CFS against *Salmonella* was not found for pH-neutralized CFS, indicating the possible existence of pH-sensitive bacteriocin-like peptides in the CFS.

HRMS study indicates *LS-ARS2* CFS consists of metabolite betaine, which is an essential osmolyte and neuroprotectant with significant health-improving roles (Arumugam et al., 2021). *LS-ARS2* CFS also consists of gamma-aminobutyric acid (GABA), one of the principal neurotransmitters. GABA significantly regulates nerve cell hyperactivity associated with anxiety, stress, convulsions, epilepsy, Parkinson’s, and Alzheimer’s diseases. Several studies have also reported the immunological and antimicrobial functions of GABA (Bs et al., 2021). Further, genomic analysis reveals the presence of CAZymes in the *LS-ARS2* genome. Bacterial strains use CAZymes for carbohydrate degradation. Therefore, CAZymes in the *LS-ARS2* not only could participate in utilizing complex carbohydrates and thrive in the host intestinal ecosystem; they also could further stimulate the growth of other probiotics by providing the carbon source for them. Therefore, as a nutritional supplement, *LS-ARS2* with CAZymes could facilitate the overall good bacterial distribution and improve gut-health.

Food supplements are added to the diet to maintain the nutritional balance of the body. HRMS analysis indicates *LS-ARS2* CFS is enriched with several beneficial metabolites that highlight the potential of the strain to be used as a dietary supplement. For instance, essential amino acids like threonine, lysine, phenylalanine, leucine, isoleucine, and valine are detected in the CFS of *LS-ARS2.* Next, vitamins nicotinic acid (vitamin B_3_) and pyridoxal (vitamin B_6_) are also found in *LS-ARS2* CFS, indicating that application of the strain could reduce the severity of clinical vitamin deficiencies (Gasperi et al., 2019; Stach et al., 2021). Moreover, *LS-ARS2* CFS is enriched with metabolites like pyruvic acid, oxaloacetate, and oxoglutaric acid, which are reported to have health-promoting effects (Dash et al., 2023; Wernerman et al., 1990).

Besides, *LS-ARS2* CFS is also enriched with metabolites important for food processing and cosmetic industries. Metabolites like citric acid, dihydroxyacetone, and acetoin have extensive applications in the poultry, food, and cosmetic industries (Dash et al., 2023). In our genomic study, antiSMASH predicts the presence of the T3PKS region, which is known to encode substances with enormous functions in food processing industries. Other detected metabolites in *LS-ARS2* CFS, such as adipic acid, can be used as food additives (Horn et al., 1957), whereas 3-Hydroxypropeonate, 2.3-butanediol, and malonic semialdehyde have diverse applications in the pharmaceutical, cosmetics, and agriculture industries (Zhao and Tian, 2021; Gu et al., 2022; Dash et al., 2023).

## 5 Conclusion

We present a full-fledged study on the probiotic attributes of *LS-ARS2*. (a) The remarkable anti-oxidant and anti-bacterial potential of *LS-ARS2* encourages further studies of the strain for health-promoting applications. (b) The strong biofilm-forming ability of *LS-ARS2* ensures efficient and longer stay in the GI tract. The significant anti-biofilm activity of *LS-ARS2* CFS for *MRSA* again suggests the use of *LS-ARS2* as a promising antimicrobial agent to inhibit pathogenic biofilm formation. (c) As a gut microbe, *LS-ARS2* could naturally re-establish the gut-microbial composition and thereby improve the metabolic status of the gut and prevent dysbiosis. Further, the identification of beneficial metabolites in *LS-ARS2-*CFS with antimicrobial and immunomodulatory properties indicates the therapeutic application of *LS-ARS2* in different diseases. However, *in vivo* studies with gut-inflammatory disease models would reaffirm that.

The potential ability of *LS-ARS2* to stably colonize in the GI tract and other probiotic attributes, including beneficial metabolic profiling of *LS-ARS2-*CFS, indicate the promising application of the strain as a food supplement. Further, myriads of secondary metabolites with industrial applications were detected in *LS-ARS2* CFS, which supports the potential use of the strain in the food as well as poultry and cosmetic industries. Together, the study indicates that *LS-ARS2* demands further studies to determine its potential application in the future.

In summary, the study offers a novel and promising probiotic strain, *LS-ARS2,* that demands *in vivo* research, particularly focusing on gut-disease models for their potential applications in the near future.

## Data Availability

The datasets presented in this study can be found in online repositories. The names of the repository/repositories and accession number(s) can be found in the article.

## Footnotes

^1^ https://www.metabolomicsworkbench.org/data/M_form.php#M2

## Glossary

GI: gastrointestinal
WHO: World Health Organization
WGS: Whole Genome Sequencing
ATCC: American Type Culture Collection
MRS: De Man Rogosa Sharpe agar
DMEM: Dulbecco’s Modified Eagle Medium
FBS: Fetal Bovine Serum
MTCC: Microbial Type Culture Collection
PBS: Phosphate Buffered Saline
MOI: Multiplicity Of Infection
SS: Salmonella Shigella
DPPH: 1,1-diphenyl-2-picrylhydrazyl
ABTS: 2,2′-azino-bis (3-ethylbenzothiazoline-6-sulfonic acid)
PCR: polymerase chain reaction
CFU: colony forming unit
ANI: Average Nucleotide Identity
CRISPR: Clustered Regularly Interspaced Short Palindromic Repeats
SCFA: short-chain fatty acid
PKs: Polyketide Synthases
NRPs: Nonribosomal peptides
RiPP: Ribosomally synthesized and post-translationally modified peptides
