# Supplementary material for: Complete genome sequence, metabolic profiling and functional studies reveal Ligilactobacillus salivarius LS-ARS2 is a promising biofilm-forming probiotic with significant antioxidant, antibacterial, and antibiofilm potential
Source: Front Microbiol. 2025 Mar 20;16:1535388. doi: 10.3389/fmicb.2025.1535388 (PMC11965632; doi:10.3389/fmicb.2025.1535388)
Supplement: Supplementary file 1 [file Data_Sheet_1.pdf]

Complete genome sequence, metabolic profiling and functional studies reveal *Ligilactobacillus salivarius LS-ARS2* is a promising biofilm-forming probiotic with significant antioxidant, antibacterial, and antibiofilm potential

Sinjini Patra[1], Biswaranjan Pradhan[2], and Anasuya Roychowdhury[1#]

Affiliations: [1]Biochemistry and Cell Biology Laboratory, School of Basic Sciences, Indian Institute of Technology Bhubaneswar, Odisha, 752050, India; [2]S. K. Dash Center of Excellence of Biosciences and Engineering & Technology (SKBET), Indian Institute of Technology Bhubaneswar, Odisha, 752050, India

Address for Correspondence: [#1]Biochemistry and Cell Biology Laboratory, School of Basic Sciences, Indian Institute of Technology Bhubaneswar, Odisha, 752050, India. Tel: +91-674-713-5106, e-mail: aroychowdhury@iitbbs.ac.in [ORCID ID: 0000-0003-3735-3021]

Supplementary Figures

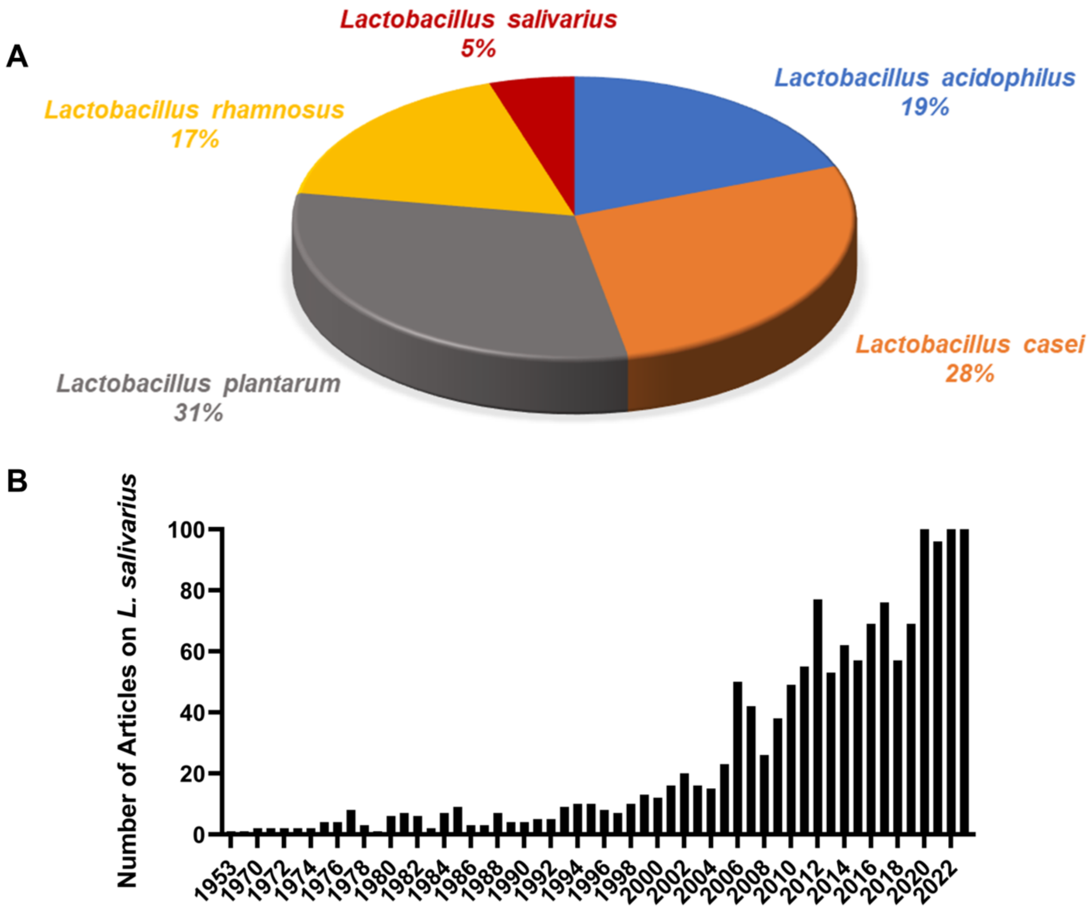

Supplementary Figure S1 Updated scenario of research on *Lactobacillus salivarius* strains. (A) Research trend showing that compared to other LAB, available studies on *Lactobacillus salivarius* strains are limited. **(B)** Number of research article published on *L. salivarius* is consistently increased showing the promising attributes of *L. salivarius.*

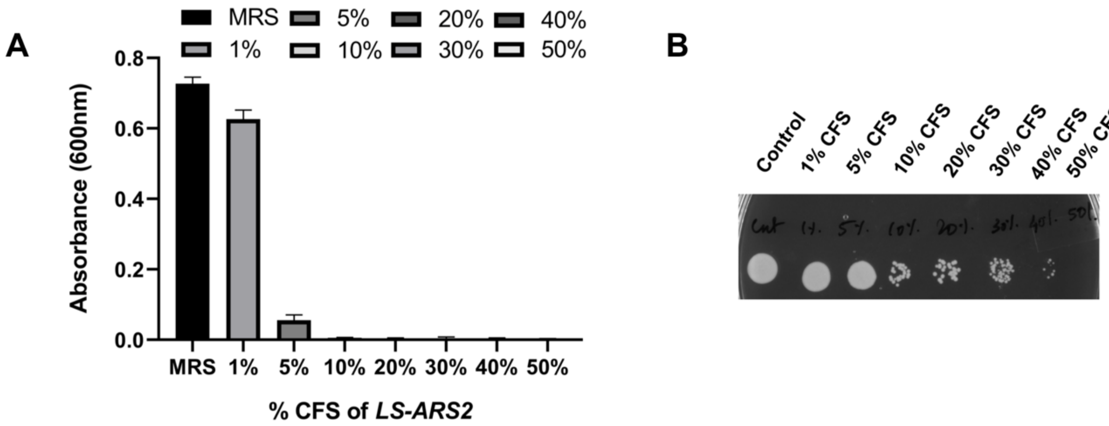

Supplementary Figure S2 Determination of minimum inhibitory percentage (MIP) of *LS-ARS2*-derived CFS for Methicillin-resistant *Staphylococcus aureus* (MRSA) (A) To determine the MIP, MRSA was grown in different percentages of *LS-ARS2* CFS. **(B) To determine the viability of MRSA**, from each well of pathogen cultured in *LS-ARS2* CFS, spotting was performed and checked whether the effect was bactericidal or bacteriostatic. All the data were represented as mean ± SD of three replicates.

A

Class	Class Description	Family (Number of genes)	Number of family	Number of genes
**CBM**	Carbohydrate-Binding Molecule	CBM48 (2), CBM50 (3)	2	5
**GH**	Glycoside Hydrolases	GH13 (5), GH31 (2), GH32 (1), GH37 (1), GH65 (2), GH77 (1)	6	12
**GT**	Glycocyl Tranferases	GT1 (1), GT2 (1), GT4 (4), GT5 (2), GT26 (1), GT28 (1), GT35 (1), GT51 (2)	8	13

B

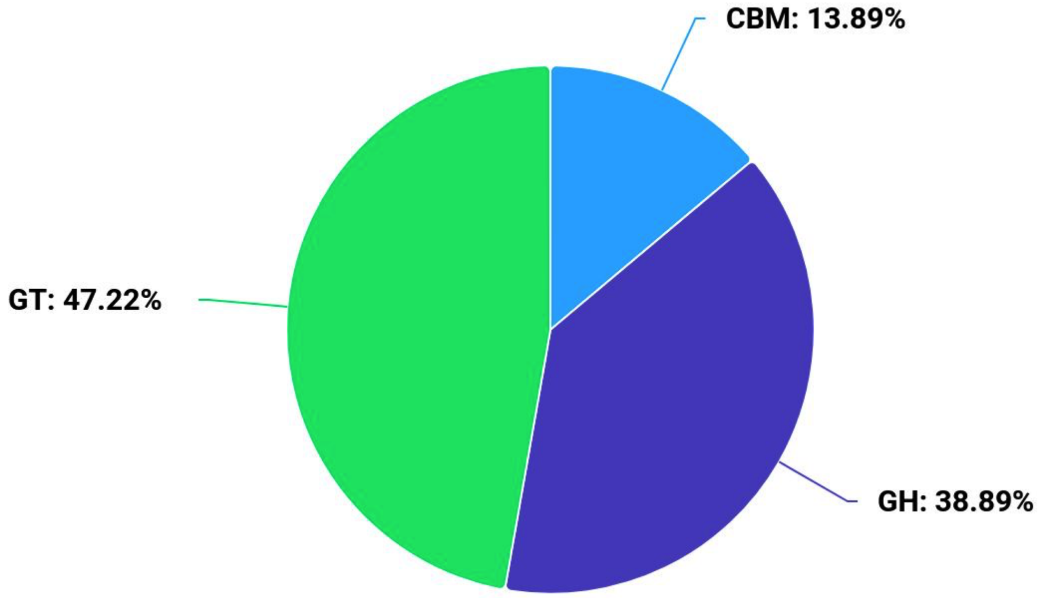

Supplementary Figure S3 Carbohydrate-activated enzymes (CAZymes) detected in the genome of *LS-ARS2.* (A) different classes and families of cazymes and the number of genes encoding the corresponding cazymes. **(B)** the distribution of cazymes in the genome of *LS-ARS2.*

Supplementary Tables

Supplementary Table S1 Overall assembly summary

Sample name	Raw data	Data after AT (Gb)	Read quality before AT	Read quality after AT	GC% before AT	GC% after AT	Coverage (>=30x)	Depth
ARS-2	1.22	0.85	37.75	37.74	35.5	33.35	90.12%	290.81

Data after AT (Gb): Data after Adapter Trimming
Read quality before AT: Read quality before Adapter Trimming
Read quality after AT: Read quality after Adapter Trimming
GC% before AT: GC% before Adapter Trimming
GC% after AT: GC% after Adapter Trimming
Coverage (%): The percentage of mapped sited (>= 1x)
Depth: Average mapping depth

Supplementary Table S2 Functions of the relevant genes obtained from the *LS-ARS2* genome

Preferred_name	Function	Feature
yugI	general stress protein	Stress tolerance
hrcA	Regulation of heat-shock induction	Heat shock regulator
ctsR	Belongs to the CtsR family	Heat shock regulator
dnaJ	Molecular chaperon	Heat shock tolerance
groS	Molecular chaperon	Heat shock tolerance
groL	Prevents misfolding and promotes refolding of polypeptides under stress conditions	Heat shock tolerance
hslO	Redox regulated molecular chaperone.	Heat shock tolerance
hslU	Molecular chaperon	Heat shock tolerance
hslV	Molecular chaperon	Heat shock tolerance
clpX	ATP-dependent chaperone functions	Heat shock tolerance
clpL	ATP-dependent chaperone functions	Heat shock tolerance
clpP	Plays a major role in the degradation of misfolded proteins	Heat shock tolerance
clpE	ATP-dependent chaperone functions	Heat shock tolerance
cspC	Cold shock protein	Stress tolerance
atpA	ATPase proton pump	Acid tolerance
atpC	ATPase proton pump	Acid tolerance
atpD	ATPase proton pump	Acid tolerance
atpE	ATPase proton pump	Acid tolerance
atpF	ATPase proton pump	Acid tolerance
atpG	ATPase proton pump	Acid tolerance
atpH	ATPase proton pump	Acid tolerance
plsC	Acyltransferase	Acid tolerance
pyk	Belongs to the pyruvate kinase family	Acid tolerance
clpC	Part of a stress-induced multi-chaperone system	Stress tolerance
asp23	Alkaline shock protein	Acid tolerance
ppaC	Inorganic pyrophosphatase	Bile tolerance
nhaC	Na H antiporter NhaC	Acid tolerance
cfa	Cyclopropane-fatty-acyl-phospholipid synthase	Bile tolerance
oppA	ABC transporter, substratebinding protein	Bile tolerance

oppB	ABC-type dipeptide oligopeptide nickel transport systems, permease components	Bile tolerance
oppC	ABC-type dipeptide oligopeptide nickel transport systems, permease components	Bile tolerance
oppD	Belongs to the ABC transporter superfamily	Bile tolerance
oppF	Belongs to the ABC transporter superfamily	Bile tolerance
yhcA	ABC transporter, ATP-binding protein	Stress tolerance
lmrA	ABC transporter, ATP-binding protein	Stress tolerance
yfiC	ABC transporter	Stress tolerance
ybhF_2	AAA domain, putative AbiEii toxin, Type IV TA system	Stress tolerance
ybhR	ABC transporter	Stress tolerance
VPA1512	Bacterial extracellular solute-binding proteins, family 3	Stress tolerance
veg	Biofilm formation stimulator VEG	Biofilm formation
argH	argininosuccinate lyase	Metabolism
glnP	ABC transporter	Bile tolerance
glnA	glutamine synthetase	Bile tolerance
glnH	ABC transporter	Bile tolerance
glnPH2	ABC transporter permease	Bile tolerance
uspA	Universal stress protein family	Stress tolerance
ytmP	Choline/ethanolamine kinase	Metabolism
ecsB	ABC transporter	Stress tolerance
grpE	Participates in the response to hyperosmotic and heat shock	Heat shock tolerance
dnaK	Heat shock 70 kDa protein	Heat shock tolerance
glnR	ABC transporter	Bile tolerance
glnQ	ABC transporter, ATP-binding protein	Bile tolerance
atpB	it plays a direct role in the translocation of protons across the membrane	Acid tolerance
clpB	Part of a stress-induced multi-chaperone system	Heat shock tolerance
natB	ABC-type Na efflux pump, permease component	Acid tolerance
dltD	Protein involved in D-alanine esterification of lipoteichoic acid and wall teichoic acid (D-alanine transfer protein)	Immunomodulation
dltC	Carrier protein involved in the D-alanylation of lipoteichoic acid (LTA).	Immunomodulation
dltB	MBOAT, membrane-bound O-acyltransferase family	Immunomodulation
dltA	Catalyzes the first step in the D-alanylation of lipoteichoic acid (LTA)	Immunomodulation
dltX	D-Ala-teichoic acid biosynthesis protein	Immunomodulation

mntH	H (+)-stimulated, divalent metal cation uptake system	Acid tolerance
nrdG	Activation of anaerobic ribonucleoside-triphosphate reductase under anaerobic conditions	Anaerobic growth
tcyB	ABC transporter	Acid tolerance
yjcE	Sodium proton antiporter	Acid tolerance
hrtB	ABC transporter permease	Acid tolerance
devA	ABC transporter, ATP-binding protein	Acid tolerance
luxS	Involved in the synthesis of autoinducer 2 (AI-2) and quorum sensing	Biofilm formation
uspA	universal stress protein	Stress tolerance
cydD	ABC transporter transmembrane region	Acid tolerance
cydD	ABC transporter	Acid tolerance
aatB	ABC transporter substrate-binding protein	Acid tolerance
fhuC	ABC transporter	Acid tolerance
znuB	ABC 3 transport family	Acid tolerance
cas3	CRISPR-associated helicase cas3	Safety attribute
casB	CRISPR-associated protein Cse2 (CRISPR_cse2)	Safety attribute
casD	CRISPR-associated protein (Cas_Cas5)	Safety attribute
casE	CRISPR_assoc	Safety attribute
cas1	Provides protection against mobile genetic elements	Safety attribute
cas2	CRISPR-associated protein (Cas_Cas2CT1978)	Safety attribute
mapA	hydrolase, family 65, central catalytic	Adhesion
lspA	This protein specifically catalyzes the removal of signal peptides from prolipoproteins	Adhesion
srtA	sortase family	Adhesion
eno	Catalyzes the reversible conversion of 2- phosphoglycerate into phosphoenolpyruvate	Adhesion
metI	ABC transporter permease	Acid tolerance
metN	Part of the ABC transporter complex MetNIQ involved in methionine import	Acid tolerance
metI	ABC transporter permease	Acid tolerance
usp6	universal stress protein	Stress tolerance
pflA	Activation of pyruvate formate-lyase under anaerobic conditions	Anaerobic growth
potD	ABC transporter	Stress tolerance
potB	ABC transporter permease	Stress tolerance

tpx	Thiol-specific peroxidase that catalyzes reduction of hydrogen peroxide and organic hydroperoxides to water and alcohols, respectively	Antioxidant
yfeX	Peroxidase	Antioxidant
trxA	Belongs to the thioredoxin family	Antioxidant
trxB	Belongs to the class-II pyridine nucleotide-disulfide oxidoreductase family	Antioxidant
yjbH	Thioredoxin	Antioxidant
gshF	Belongs to the glutamate--cysteine ligase type 1 family	Antioxidant
ndh	NADH dehydrogenase	Antioxidant
npr	Pyridine nucleotide-disulphide oxidoreductase, dimerisation domain	Antioxidant
nrdH	Glutaredoxin	Antioxidant
msrA	Catalyzes the reversible oxidation-reduction of methionine sulfoxide in proteins to methionine and protects proteins from oxidation	Antioxidant
msrB	peptide methionine sulfoxide reductase	Antioxidant
pts13C	The phosphoenolpyruvate-dependent sugar phosphotransferase system (PTS)	Sugar transport
hprK	Participate in sugar transport by sugar phosphotransferase system (PTS)	Sugar transport
ptsH	Phosphocarrier protein HPR	Sugar transport
ptsI	General component of the phosphoenolpyruvate-dependent sugar PTS system	Sugar transport
epsL	Bacterial sugar transferase	Sugar transport
mtlF	Sugar translocation across the cell membrane	Sugar transport
ptsI	Phosphoenolpyruvate-dependent sugar PTS	Sugar transport
uxuT	MFS/sugar transport protein	Sugar transport
glcU	Sugar transport	Sugar transport
rfbP	Bacterial sugar transferase	Sugar transport
manL	PTS system sorbose subfamily IIB component	Sugar transport
dhaM	PTS system fructose IIA component	Sugar transport
srlB	PTS system glucitol/sorbitol-specific IIA component	Sugar transport
dhaK	Dak1 domain	Sugar transport
dhaL	Dak2	Sugar transport
srlA	PTS system enzyme II sorbitol-specific factor	Sugar transport
mtlA	PTS system, Lactose/Cellobiose specific IIB subunit	Sugar transport
srlE	Sorbitol phosphotransferase enzyme II N-terminus	Sugar transport
mtlD	Mannitol-1-phosphate 5-dehydrogenase activity	Sugar transport

mtlR	Mga helix-turn-helix domain	Sugar transport
srlM	Mga helix-turn-helix domain	Sugar transport
manY	PTS system sorbose-specific iic component	Sugar transport
manA	Mannose-6-phosphate isomerase	Sugar transport
manN	PTS system sorbose subfamily IIB component	Sugar transport
comA	ABC-type bacteriocin lantibiotic exporters	Bacteriocin transport
pta	phosphate acetyltransferase	Fermentation
ldhD	Belongs to the D-isomer specific 2-hydroxyacid dehydrogenase family	Fermentation
ldh	Belongs to the LDH MDH superfamily. LDH family	Fermentation
ldhA	D-isomer specific 2-hydroxyacid dehydrogenase, catalytic domain	Fermentation
adhE	belongs to the iron- containing alcohol dehydrogenase family	Fermentation
ackA	Catalyzes the formation of acetyl phosphate from acetate and ATP	Fermentation
xfp	Phosphoketolase	Fermentation
sfcA	Malic enzyme	Fermentation
nagA	Belongs to the metallo-dependent hydrolases superfamily. NagA family	SCFA Biosynthesis
acyP	Belongs to the acylphosphatase family	SCFA Biosynthesis
lpdA	Dehydrogenase	SCFA Biosynthesis
pdhC	Dihydrolipoamide acetyltransferase component of pyruvate dehydrogenase complex	SCFA Biosynthesis
pdhB	Transketolase, C-terminal domain protein	SCFA Biosynthesis
pdhA	Dehydrogenase E1 component	SCFA Biosynthesis
fabI	Enoyl- acyl-carrier-protein reductase NADH	SCFA Biosynthesis
accD	Component of the acetyl coenzyme A carboxylase (ACC) complex	SCFA Biosynthesis
accC	Acetyl-CoA carboxylase biotin carboxylase subunit	SCFA Biosynthesis
accB	Involved in the production of malonyl-CoA	SCFA Biosynthesis
fabD	Malonyl CoA-acyl carrier protein transacylase	SCFA Biosynthesis
fabH	Catalyzes the condensation reaction of fatty acid synthesis	SCFA Biosynthesis
eda	KDPG and KHG aldolase	SCFA Biosynthesis
poxB	Belongs to the TPP enzyme family	SCFA Biosynthesis
patB	Aminotransferase, class I	SCFA Biosynthesis
malY	Aminotransferase, class I	SCFA Biosynthesis
alsS	Belongs to the TPP enzyme family	SCFA Biosynthesis
pyk	Belongs to the pyruvate kinase family	SCFA Biosynthesis

gabD	Belongs to the aldehyde dehydrogenase family	SCFA Biosynthesis
argE	succinyl-diaminopimelate desuccinylase	SCFA Biosynthesis
sfcA	Malic enzyme	SCFA Biosynthesis
yncA	Maltose acetyltransferase	SCFA Biosynthesis
fadB4	3-hydroxyacyl-CoA dehydrogenase	SCFA Biosynthesis
mvaA	Belongs to the HMG-CoA reductase family	SCFA Biosynthesis
glxR	Dehydrogenase	SCFA Biosynthesis
mvaA	Belongs to the HMG-CoA reductase family	SCFA Biosynthesis
adk	Plays an important role in adenine nucleotide metabolism	Vitamin metabolism
thiN	thiamine pyrophosphokinase	Vitamin metabolism
thiT	Thiamine transporter protein (Thia_YuaJ)	Vitamin metabolism
thiI	Thiamine biosynthesis pathway	Vitamin metabolism
thiD	Phosphomethylpyrimidine kinase	Vitamin metabolism
thiJ	DJ-1/PfpI family	Vitamin metabolism
pdxK	Phosphomethylpyrimidine kinase	Vitamin metabolism
birA	Acts both as a biotin-- acetyl-CoA-carboxylase ligase and a repressor	Vitamin metabolism
folA	Key enzyme in folate metabolism	Vitamin metabolism
folC	Belongs to the folylpolyglutamate synthase family	Vitamin metabolism
folD	Catalyzes synthesis of 10- formyltetrahydrofolate	Vitamin metabolism
ycsE	Sucrose-6F-phosphate phosphohydrolase	Vitamin metabolism
yitU	hydrolase	Vitamin metabolism
rsgA	Helps release RbfA from mature subunits	Vitamin metabolism
engB	Necessary for normal cell division and for the maintenance of normal septation	Vitamin metabolism
iscS	Aminotransferase class V	Vitamin metabolism
nudF	ADP-ribose pyrophosphatase	Vitamin metabolism
nfrA	NADPH-dependent FMN reductase	Vitamin metabolism
adhE	belongs to the iron- containing alcohol dehydrogenase family	Vitamin metabolism
ribF	Belongs to the ribF family	Vitamin metabolism
fabI	Enoyl- acyl-carrier-protein reductase NADH	Vitamin metabolism
fabZ	Involved in unsaturated fatty acids biosynthesis	Vitamin metabolism
fabF	Catalyzes the condensation reaction of fatty acid synthesis involving malonyl-ACP	Vitamin metabolism
fabD	Malonyl CoA-acyl carrier protein transacylase	Vitamin metabolism

fabH	Role in fatty acid synthesis governing the total rate of fatty acid production	Vitamin metabolism
nadD	Catalyzes the reversible adenylation of nicotinate mononucleotide (NaMN) to nicotinic acid adenine dinucleotide (NaAD)	Vitamin metabolism

Supplementary Table S3 Predicted prophage regions within the genome of *LS-ARS2*

Contig	Region	Region Length (kb)	Completeness	Score	Total Protein	Region Position	Most Common Phage	GC%
28	1	8.4	Incomplete	10	8	352-8848	PHAGE_Prochl_P_SSM2_NC_006883(4)	33.73
48	1	10	Incomplete	10	12	27884-37902	PHAGE_Entero_phiEF24C_NC_00904(3)	31.48
	2	7.9	Incomplete	10	8	41090-49082	PHAGE_Cellul_phi38:1_NC_021796(1)	32.50
77	1	9.1	Incomplete	10	8	49876-59038	PHAGE_Bacill_G_NC_023719(2)	34.30

Supplementary Table S4 CRISPR-Cas arrays in the *LS-ARS2* genome

Element	CRISPR Id / Cas Type	Start	End	Spacer / Gene	Repeat consensus / cas genes
Cas cluster	CAS	10790	20037	7	cas2_TypeIE, cas3_TypeI, cas3_TypeI, cas5_TypeIE, cas6_TypeIE, cas7_TypeIE, cse2_TypeIE
Cas cluster	CAS-TypeIE	15249	20037	6	cas1_TypeIE, cas2_TypeIE, cas5_TypeIE, cas6_TypeIE, cas7_TypeIE, cse2_TypeIE
Cas cluster	CAS	23110	25140	2	cas3_TypeI, cas3_TypeI
Cas cluster	CAS	7729	9051	2	cas3_TypeI, cas3_TypeI
CRISPR	JVAF01000101_1_1	56	146	1	AAAGTAATACCAATCGTTACCACCTTG
CRISPR	JVAF01000101_1_2	245	394	2	AAAGTAATACCAATCGTTACCACCTTG
CRISPR	JVAF01000103_1_1	19887	19978	1	TTCAATCCAACAAGTGGACGTATGCAAAA
Cas cluster	CAS	37512	49026	3	cas4_TypeI-II, cas3_TypeI, cas3_TypeI

Supplementary Table S5 Antibiotic resistance gene family, drug class and resistance mechanism of *LS-ARS2*

RGI criteria	ARO term	Detection criteria	AMR gene family	Drug class	Resistance mechanism	% Identity of Matching region	% Length of Reference Sequence
Strict	vanT gene in vanG cluster	protein homolog model	glycopeptide resistance gene cluster, vanT	glycopeptide antibiotic	antibiotic target alteration	35.52	52.11

Supplementary Table S6 Overview of the KEGG pathways detected in *LS-ARS2* genome

KEGG category	KEGG Functional Class	Mapped Objects
Metabolism	Carbohydrate metabolism	179
Metabolism	Energy metabolism	56
Metabolism	Lipid metabolism	37
Metabolism	Nucleotide metabolism	56
Metabolism	Amino acid metabolism	94
Metabolism	Metabolism of other amino acids	27
Metabolism	Glycan biosynthesis and metabolism	35
Metabolism	Metabolism of cofactors and vitamins	55
Metabolism	Metabolism of terpenoids and polyketides	17
Metabolism	Biosynthesis of other secondary metabolites	23
Metabolism	Xenobiotics biodegradation and metabolism	18
Genetic Information Processing	Transcription	6
Genetic Information Processing	Translation	79
Genetic Information Processing	Folding, sorting and degradation	23
Genetic Information Processing	Replication and repair	61
Environmental Information Processing	Membrane transport	62
Environmental Information Processing	Signal transduction	36
Cellular Processes	Transport and catabolism	2
Cellular Processes	Cell growth and death	12
Cellular Processes	Cellular community - prokaryotes	23
Cellular Processes	Cell motility	4
Organismal Systems	Immune system	1
Organismal Systems	Endocrine system	12
Organismal Systems	Digestive system	6
Organismal Systems	Nervous system	2
Organismal Systems	Aging	4
Organismal Systems	Environmental adaptation	2
Human Diseases	Cancer	11
Human Diseases	Infectious disease: viral	1
Human Diseases	Infectious disease: bacterial	13
Human Diseases	Infectious disease: parasitic	1
Human Diseases	Immune disease	1
Human Diseases	Neurodegenerative disease	2

Human Diseases	Cardiovascular disease	8
Human Diseases	Endocrine and metabolic disease	5
Human Diseases	Drug resistance: antimicrobial	20
Human Diseases	Drug resistance: antineoplastic	5

Supplementary Table S7 KEGG pathways detected in the genome of *LS-ARS2*

KEGG Pathways	Mapped objects
**Global and overview maps**	
01100 Metabolic pathways	298
01110 Biosynthesis of secondary metabolites	136
01120 Microbial metabolism in diverse environments	70
01200 Carbon metabolism	41
01210 2-Oxocarboxylic acid metabolism	9
01212 Fatty acid metabolism	10
01230 Biosynthesis of amino acids	48
01232 Nucleotide metabolism	28
01250 Biosynthesis of nucleotide sugars	20
01240 Biosynthesis of cofactors	51
01220 Degradation of aromatic compounds	3
**Carbohydrate metabolism**	**179**
00010 Glycolysis / Gluconeogenesis	21
00020 Citrate cycle (TCA cycle)	7
00030 Pentose phosphate pathway	15
00040 Pentose and glucuronate interconversions	5
00051 Fructose and mannose metabolism	18
00052 Galactose metabolism	13
00500 Starch and sucrose metabolism	18
00520 Amino sugar and nucleotide sugar metabolism	23
00620 Pyruvate metabolism	22
00630 Glyoxylate and dicarboxylate metabolism	7
00640 Propanoate metabolism	10
00650 Butanoate metabolism	7
00660 C5-Branched dibasic acid metabolism	2
00562 Inositol phosphate metabolism	2
**Energy metabolism**	**56**
00190 Oxidative phosphorylation	12
00195 Photosynthesis	8
00710 Carbon fixation by Calvin cycle	8
00720 Carbon fixation pathways	10
00680 Methane metabolism	11
00910 Nitrogen metabolism	2
00920 Sulfur metabolism	5
**Lipid metabolism**	**37**
00061 Fatty acid biosynthesis	10
00071 Fatty acid degradation	1
00074 Mycolic acid biosynthesis	2
00120 Primary bile acid biosynthesis	1
00121 Secondary bile acid biosynthesis	1
00561 Glycerolipid metabolism	12
00564 Glycerophospholipid metabolism	8
00600 Sphingolipid metabolism	2
**Nucleotide metabolism**	**56**
00230 Purine metabolism	34
00240 Pyrimidine metabolism	22

**Amino acid metabolism**	**94**
00250 Alanine, aspartate and glutamate metabolism	19
00260 Glycine, serine and threonine metabolism	13
00270 Cysteine and methionine metabolism	21
00280 Valine, leucine and isoleucine degradation	4
00290 Valine, leucine and isoleucine biosynthesis	2
00300 Lysine biosynthesis	13
00310 Lysine degradation	3
00220 Arginine biosynthesis	7
00330 Arginine and proline metabolism	6
00350 Tyrosine metabolism	2
00360 Phenylalanine metabolism	2
00380 Tryptophan metabolism	1
00400 Phenylalanine, tyrosine and tryptophan biosynthesis	1
**Metabolism of other amino acids**	**27**
00430 Taurine and hypotaurine metabolism	3
00450 Selenocompound metabolism	5
00460 Cyanoamino acid metabolism	3
00470 D-Amino acid metabolism	11
00480 Glutathione metabolism	5
**Glycan biosynthesis and metabolism**	**35**
00603 Glycosphingolipid biosynthesis - globo and isoglobo series	1
00511 Other glycan degradation	1
00540 Lipopolysaccharide biosynthesis	1
00542 O-Antigen repeat unit biosynthesis	1
00550 Peptidoglycan biosynthesis	18
00552 Teichoic acid biosynthesis	11
00572 Arabinogalactan biosynthesis - Mycobacterium	1
00543 Exopolysaccharide biosynthesis	1
**Metabolism of cofactors and vitamins**	**55**
00730 Thiamine metabolism	7
00740 Riboflavin metabolism	4
00750 Vitamin B6 metabolism	3
00760 Nicotinate and nicotinamide metabolism	6
00770 Pantothenate and CoA biosynthesis	8
00780 Biotin metabolism	5
00785 Lipoic acid metabolism	5
00790 Folate biosynthesis	2
00670 One carbon pool by folate	11
00130 Ubiquinone and other terpenoid-quinone biosynthesis	4
**Metabolism of terpenoids and polyketides**	**17**
00900 Terpenoid backbone biosynthesis	10
00908 Zeatin biosynthesis	1
00523 Polyketide sugar unit biosynthesis	4
01054 Nonribosomal peptide structures	1
01055 Biosynthesis of vancomycin group antibiotics	1
**Biosynthesis of other secondary metabolites**	**23**
00966 Glucosinolate biosynthesis	1

00311 Penicillin and cephalosporin biosynthesis	1
00332 Carbapenem biosynthesis	2
00261 Monobactam biosynthesis	4
00521 Streptomycin biosynthesis	7
00524 Neomycin, kanamycin and gentamicin biosynthesis	1
00525 Acarbose and validamycin biosynthesis	2
00333 Prodigiosin biosynthesis	3
00998 Biosynthesis of various antibiotics; Including: Kanosamine biosynthesis, Aurachin biosynthesis, Bacilysin biosynthesis, Puromycin biosynthesis, Dapdiamides biosynthesis, Fosfomycin biosynthesis, Cremeomycin biosynthesis, Fumagillin biosynthesis, Pentalenolactone biosynthesis, Terpentecin biosynthesis, Roseoflavin biosynthesis, Cycloserine biosynthesis	1
00999 Biosynthesis of various plant secondary metabolites; Including: Crocin biosynthesis, Ginsenoside biosynthesis, Saponin adjuvant biosynthesis, Cannabidiol biosynthesis, Mugineic acid biosynthesis, Pentagalloylglucose biosynthesis, Benzoxazinoid biosynthesis, Gramine biosynthesis, Coumarin biosynthesis, Furanocoumarin biosynthesis, Hordatine biosynthesis, Podophyllotoxin biosynthesis	1
**Xenobiotics biodegradation and metabolism**	**18**
00362 Benzoate degradation	3
00627 Aminobenzoate degradation	1
00625 Chloroalkane and chloroalkene degradation	2
00361 Chlorocyclohexane and chlorobenzene degradation	1
00622 Xylene degradation	1
00621 Dioxin degradation	1
00626 Naphthalene degradation	1
00983 Drug metabolism - other enzymes	8
**Transcription**	**6**
03020 RNA polymerase	5
03022 Basal transcription factors	1
**Translation**	**79**
03010 Ribosome	53
00970 Aminoacyl-tRNA biosynthesis	25
03008 Ribosome biogenesis in eukaryotes	1
**Folding, sorting and degradation**	**23**
03060 Protein export	10
04122 Sulfur relay system	3
03018 RNA degradation	10
**Replication and repair**	**61**
03030 DNA replication	13
03410 Base excision repair	7
03420 Nucleotide excision repair	7
03430 Mismatch repair	16
03440 Homologous recombination	18
**Membrane transport**	**62**
02010 ABC transporters	38

02060 Phosphotransferase system (PTS)	16
03070 Bacterial secretion system	8
**Signal transduction**	**36**
02020 Two-component system	23
04016 MAPK signaling pathway - plant	2
04066 HIF-1 signaling pathway	7
04070 Phosphatidylinositol signaling system	2
04152 AMPK signaling pathway	1
04075 Plant hormone signal transduction	1
**Transport and catabolism**	**2**
04146 Peroxisome	1
04148 efferocytosis	1
**Cell growth and death**	**12**
04112 Cell cycle - Caulobacter	10
04217 Necroptosis	2
**Cellular community - prokaryotes**	**23**
02024 Quorum sensing	17
05111 Biofilm formation - Vibrio cholerae	2
02026 Biofilm formation - Escherichia coli	4
**Cell motility**	**4**
02030 Bacterial chemotaxis	1
02040 Flagellar assembly	2
04820 Cytoskeleton in muscle cells	1
**Immune system**	**1**
04621 NOD-like receptor signaling pathway	1
**Endocrine system**	**12**
04910 Insuline signaling pathway	1
04922 Glucagon signaling pathway	7
03320 PPAR signaling pathway	2
04917 Prolactin signaling pathway	1
04918 Thyroid hormone signaling pathway	1
**Digestive system**	**6**
04981 Folate transport and metabolism	5
04978 Mineral absorption	1
**Nervous system**	**2**
04724 Glutamatergic synapse	1
04727 GABAergic synapse	1
**Aging**	**4**
04212 Longevity regulating pathway - worm	3
04213 Longevity regulating pathway - multiple species	1
**Environmental adaptation**	**2**
04626 Plant-pathogen interaction	2
**Cancer**	**11**
05200 Pathways in cancer	1
05205 Proteoglycans in cancer	1
05203 Viral carcinogenesis	1
05230 Central carbon metabolism in cancer	7
05211 Renal cell carcinoma	1
**Infectious disease: viral**	**1**

05165 Human papillomavirus infection	1
**Infectious disease: bacterial**	**13**
05130 Pathogenic Escherichia coli infection	1
05132 Salmonella infection	2
05134 Legionellosis	2
05150 Staphylococcus aureus infection	5
05152 Tuberculosis	3
**Infectious disease: parasitic**	**1**
05146 Amoebiasis	1
**Immune disease**	**1**
05340 Primary immunodeficiency	1
**Neurodegenerative disease**	**2**
05010 Alzheimer disease	1
05012 Parkinson disease	1
**Cardiovascular disease**	**8**
05417 Lipid and atherosclerosis	1
05418 Fluid shear stress and atherosclerosis	2
05415 Diabetic cardiomyopathy	5
**Endocrine and metabolic disease**	**5**
04930 Type II diabetes mellitus	1
04940 Type I diabetes mellitus	1
04931 Insulin resistance	2
04934 Cushing syndrome	1
**Drug resistance: antimicrobial**	**20**
01501 beta-Lactam resistance	7
01502 Vancomycin resistance	6
01503 Cationic antimicrobial peptide (CAMP) resistance	7
**Drug resistance: antineoplastic**	**5**
01524 Platinum drug resistance	1
01523 Antifolate resistance	4

Supplementary Table S8 Complete pathway modules detected in the genome of *LS-ARS2*

Carbohydrate metabolism

Central carbohydrate metabolism

M00001 Glycolysis (Embden-Meyerhof pathway), glucose => pyruvate (10) (complete 9/9)

M00002 Glycolysis, core module involving three-carbon compounds (6) (complete 5/5)

M00003 Gluconeogenesis, oxaloacetate => fructose-6P (8) (complete 7/7)

M00307 Pyruvate oxidation, pyruvate => acetyl-CoA (4) (complete 1/1)

M00006 Pentose phosphate pathway, oxidative phase, glucose 6P => ribulose 5P (3) (complete 2/2)

M00005 PRPP biosynthesis, ribose 5P => PRPP (1) (complete 1/1)

Other carbohydrate metabolism

M00061 D-Glucuronate degradation, D-glucuronate => pyruvate + D-glyceraldehyde 3P (5) (complete 5/5)

M00632 Galactose degradation, Leloir pathway, galactose => alpha-D-glucose-1P (4) (complete 4/4)

M00854 Glycogen biosynthesis, glucose-1P => glycogen/starch (4) (complete 2/2)

M00549 Nucleotide sugar biosynthesis, glucose => UDP-glucose (3) (complete 3/3)

M00554 Nucleotide sugar biosynthesis, galactose => UDP-galactose (2) (complete 2/2)

M00909 UDP-N-acetyl-D-glucosamine biosynthesis, prokaryotes, glucose => UDP-GlcNAc (5) (complete 5/5)

M00793 dTDP-L-rhamnose biosynthesis, glucose-1P => dTDP-L-Rha (4) (complete 3/3)

Energy metabolism

Carbon fixation

M00579 Phosphate acetyltransferase-acetate kinase pathway, acetyl-CoA => acetate (2) (complete 2/2)

ATP synthesis

M00157 F-type ATPase, prokaryotes and chloroplasts (8) (complete 1/1)

Lipid metabolism

Fatty acid metabolism

M00082 Fatty acid biosynthesis, initiation (6) (complete 2/2)

M00083 Fatty acid biosynthesis, elongation (4) (complete 1/1)

Nucleotide metabolism

Purine metabolism

M00048 De novo purine biosynthesis, PRPP + glutamine => IMP (12) (complete 8/8)

M00049 Adenine ribonucleotide biosynthesis, IMP => ADP,ATP (4) (complete 4/4)

M00050 Guanine ribonucleotide biosynthesis, IMP => GDP,GTP (4) (complete 4/4)

M00053 Deoxyribonucleotide biosynthesis, ADP/GDP/CDP/UDP => dATP/dGTP/dCTP/dUTP (3) (complete 2/2)

Pyrimidine metabolism

M00052 Pyrimidine ribonucleotide biosynthesis, UMP => UDP/UTP,CDP/CTP (3) (complete 3/3)
M00938 Pyrimidine deoxyribonucleotide biosynthesis, UDP => dTTP (6) (complete 5/5)

Amino acid metabolism

Serine and threonine metabolism
M00018 Threonine biosynthesis, aspartate => homoserine => threonine (5) (complete 5/5)

Cysteine and methionine metabolism
M00021 Cysteine biosynthesis, serine => cysteine (2) (complete 2/2)

Lysine metabolism
M00525 Lysine biosynthesis, acetyl-DAP pathway, aspartate => lysine (9) (complete 9/9)

Arginine and proline metabolism
M00015 Proline biosynthesis, glutamate => proline (3) (complete 2/2)

Metabolism of cofactors and vitamins

Cofactor and vitamin metabolism
M00120 Coenzyme A biosynthesis, pantothenate => CoA (4) (complete 3/3)
M00140 C1-unit interconversion, prokaryotes (3) (complete 3/3)

Biosynthesis of terpenoids and polyketides

Terpenoid backbone biosynthesis
M00364 C10-C20 isoprenoid biosynthesis, bacteria (2) (complete 2/2)

Supplementary Table S9 Identified genes within the T3PKS secondary metabolite biosynthetic gene clusters with antiSMASH

Name	Category	Function	*Score	**E-value
T3PKS: Chal_sti_synt_N SMCOG1043: hydroxymethylglutaryl-CoA synthase	Biosynthetic	Cholesterol biosynthesis	527.6	2.9e-160
SMCOG1063: argininosuccinate lyase/adenylosuccinate lyase	Biosynthetic additional	Amino acid biosynthesis	226.7	9.4e-69
ATP-grasp		Bacterial cell wall biosynthesis		
SMCOG1182: Polyprenyl synthetase		Secondary metabolites (isoprenoids)	238.9	1.1e-72
SMCOG1008: response regulator	Regulatory	Stress adaption of bacteria	205.9	9.7e-63
SMCOG1003: sensor histidine kinase			198.8	3.4e-60

*Score and **E-value: Indicates the quality of match between the *LS-ARS2* gene sequences with the reference sequences in the database
